# Macroscopic fractal dynamics characterize the “physical-metabolic” dual barriers and systemic immune exhaustion associated with primary resistance to immunotherapy in liver metastases

**DOI:** 10.3389/fimmu.2026.1878195

**Published:** 2026-07-09

**Authors:** Ziyi Guo, Chao Guo, Zhengran Lu, Kailin Zou, Hongnan Zhang

**Affiliations:** 1 Department of Nuclear Medicine, First Affiliated Hospital of Jinzhou Medical University, Jinzhou, China; 2 Architecture and Civil Engineering Institute, Guangdong University of Petrochemical Technology, Maoming, China

**Keywords:** fractal dynamics, immune checkpoint inhibitors, liver metastases, systemic immune exhaustion, tumor immune microenvironment

## Abstract

**Background:**

Liver metastases are associated with systemic immune tolerance and primary resistance to immune checkpoint inhibitors (ICIs) by establishing complex physical and metabolic barriers within the tumor immune microenvironment (TIME). We developed a non-invasive macroscopic fractal dynamics framework to map these microenvironmental barriers across scales, aiming to predict ICI efficacy in colorectal cancer liver metastases (CRLM) and lung squamous cell carcinoma (SCC).

**Methods:**

This single-center, retrospective, proof-of-concept cohort study consecutively enrolled 472 patients with CRLM or SCC liver metastases. Patients were divided into a training cohort (n=400, 2019–2024) and an independent validation cohort (n=72, 2025). Vascular fractal acceleration (*A_fd_*) and metabolic fractal dimension (*D_f_*) were extracted from contrast-enhanced magnetic resonance imaging (CE-MRI) and ^18^F-FDG PET, respectively. To eliminate baseline histological confounding, macroscopic fractal probes were Z-score normalized strictly within their respective histological cohorts. Cross-scale validation utilized digital pathology and platelet-poor plasma (PPP) cytokine profiling. An extreme gradient boosting (XGBoost) model was explicitly trained to predict a composite “High TIME Barrier” phenotype—defined by restricted CD8^+^ infiltration and low PD-L1 expression—to generate the Immuno-Radiomics Joint Score (IRJS). An exploratory survival analysis was subsequently conducted to evaluate its association with progression-free survival (PFS) among the 185 patients receiving ICI therapy. We evaluated early dynamic drift (Δ*A_fd_*) at week 3 for its utility in tracking physical barrier remodeling.

**Results:**

CRLM and SCC displayed distinct fractal trajectories indicative of metabolic and physical barriers, respectively. High *A_fd_* correlated with dense fibrovascular stroma and severe spatial exclusion of CD8^+^ T cells. High *D_f_* corresponded to severe hypoxia, CD163-enriched macrophage infiltration, and systemic immune exhaustion, characterized by elevated circulating TGF-β and exhausted IFN-γ. The IRJS demonstrated strong diagnostic performance for the High TIME barrier phenotype (temporal validation AUC: 0.912). In the ICI sub-cohort, multivariable Cox regression confirmed that an increase in the continuous baseline IRJS was a robust, independent risk factor associated with primary ICI resistance and shorter PFS.

**Conclusions:**

Macroscopic fractal dynamics offer a non-invasive, cross-scale method to evaluate the “physical-metabolic” dual microenvironmental barriers in liver metastases. The combined IRJS and dynamic Δ*A_fd_* tracking system show potential as exploratory, non-invasive surrogates to identify the systemic immune exhaustion phenotype. Pending external multi-center validation, these tools may generate hypotheses for associating macroscopic spatial barriers with primary ICI resistance and informing multidisciplinary interventions.

## 1 Introduction

Patients with solid tumor liver metastases face a generally poor clinical prognosis (1). The liver functions as an anatomical target organ for metastasis while also being closely associated with systemic immune suppression (2, 3). Remodeling of the tumor immune microenvironment (TIME) within these metastases correlates with the exhaustion of activated peripheral blood T cells, which is linked to primary resistance to immune checkpoint inhibitors (ICIs) (4). Accurately and non-invasively assessing TIME heterogeneity in liver metastases is thus critical for optimizing systemic immunotherapy in advanced tumors.

The TIME constitutes a complex system of physical and biochemical barriers (5). In advanced malignancies such as colorectal cancer liver metastases (CRLM) and lung squamous cell carcinoma liver metastases (SCC), immune escape primarily involves two spatial barriers. The “physical barrier,” formed by dense fibrotic stroma and abnormal neovasculature, sequesters effector T cells at the invasive tumor margin to create an “immune-excluded” phenotype (6, 7). The “metabolic barrier,” facilitated by hypoxia and glycolytic competition, polarizes tumor-associated macrophages (TAMs) toward an immunosuppressive CD163-enriched phenotype, suppressing intratumoral T cell function (8, 9).

Clinical assessment of these microenvironmental features heavily relies on biopsy. Single punctures, however, carry inherent spatial sampling bias and are impractical for repeated dynamic longitudinal monitoring during treatment. Medical imaging provides a non-invasive alternative for panoramic TIME assessment. Because tumor microvascular angiogenesis and metabolic reprogramming are highly nonlinear, traditional radiomics—reliant on first-order statistics or linear textural features—fail to capture complex biophysical tumor evolution. Fractal geometry offers a mathematical framework to quantify the spatial complexity of structures like distorted vascular networks and heterogeneous metabolic consumption (10). Fractal dynamics derived from this geometry quantify the self-similarity and disorder of complex systems across multiple spatial scales.

This proof-of-concept study proposes a “tumor immuno-radiomics” framework integrating macroscopic fractal dynamics from dual-modality imaging. We hypothesize that the tumor microvascular perfusion fractal parameter (*A_fd_*) from contrast-enhanced magnetic resonance imaging (CE-MRI) and the metabolic fractal dimension (*D_f_*) from positron emission tomography (PET) map stromal physical resistance and biochemical competition across scales. Consequently, these metrics may serve as non-invasive macroscopic surrogates for the TIME’s “physical barrier” and “metabolic barrier,” respectively.

We conducted a large-sample, single-center, retrospective proof-of-concept (PoC) cohort study. Using CRLM and SCC liver metastasis cohorts to model the “metabolic/physical” dual immune barriers, our objectives were threefold: (1) to map the relationships between macroscopic multimodal fractal dynamics, microscopic pathological and immunohistochemical correlates of physical and metabolic barriers, and peripheral systemic immune exhaustion; (2) to construct an immune-radiomics joint score (IRJS) by directly targeting immunohistochemistry-defined microenvironmental barriers, thereby stripping away histological confounders, and conduct an exploratory survival analysis to evaluate its association with primary ICI resistance in an independent temporal validation cohort of CRLM and SCC; and (3) to evaluate the clinical utility of early dynamic fractal drift (Δ*A_fd_*) on single-modality CE-MRI for non-invasively tracking physical barrier remodeling by targeted agents, potentially providing quantitative metrics for multidisciplinary team (MDT) interventions during the vascular normalization window.

## 2 Materials and methods

### 2.1 Study design and cohort rationale

This single-center, retrospective observational cohort study complied with the TRIPOD (Transparent Reporting of a Multivariable Prediction Model for Individual Prognosis or Diagnosis) guidelines. We consecutively enrolled 472 patients diagnosed with liver metastases, comprising 195 with colorectal cancer liver metastases (CRLM) and 277 with lung squamous cell carcinoma (SCC) liver metastases, as shown in **Supplementary flowchart**. For clarity, within this manuscript, the abbreviation ‘SCC’ strictly denotes ‘lung squamous cell carcinoma liver metastases’ unless specifically referring to the primary tumor origin, which is explicitly stated.

Consistent with clinical standards for systemic evaluation in advanced tumors, all patients underwent ^18^F-FDG PET/CT for systemic staging and primary tumor identification, alongside CE-MRI for qualitative radiological assessment of target intrahepatic lesions. Baseline core biopsy or surgical resection specimens were required for immunohistochemical (IHC) analysis.

The Independent Clinical Research Ethics Committee of the First Affiliated Hospital of Jinzhou Medical University approved the study protocol (Approval No: 2026LL-KY-063), which adhered to the ethical principles of the Declaration of Helsinki. The ethics committee waived the requirement for informed consent for the retrospective historical cohort (2019–2024). For the 2025 independent validation cohort and the platelet-poor plasma (PPP) samples utilized for cytokine detection, patients provided written informed consent prior to enrollment and blood collection. These samples were sourced from the hospital’s prospectively constructed standardized tumor biobank.

#### 2.1.1 Rationale for endpoint decoupling

CRLM and SCC liver metastases differ significantly at baseline regarding clinical stage, performance status, and prognosis. These two cohorts were analyzed collectively to represent two distinct biological paradigms colonizing the hepatic immune microenvironment: CRLM illustrating “metabolic exhaustion-mediated immune tolerance” and SCC representing “physical exclusion-mediated immune evasion”. Consequently, the primary study endpoint was strictly decoupled from clinical stage. The focus remained on validating the predictive accuracy of macroscopic multimodal imaging for the cross-scale mapping of microscopic IHC status (e.g., CD8^+^, CD31^+^, and CD163^+^ expression). Follow-up prognostic analyses employed a stratified design and were conducted independently within each disease subgroup.

#### 2.1.2 Standard temporal validation design

A rigorous temporal split was implemented to validate the robustness and real-world generalizability of the machine learning model in feature extraction and nonlinear mapping:

- Large-sample training cohort: Data collected over 5.5 years, from June 2019 to December 2024, constituted the benchmark set for model training. This extensive dataset provided a foundation for mitigating overfitting and stabilizing features.
- Independent temporal validation cohort: Continuous real-world data from January to December 2025 served strictly as the independent temporal validation set to simulate actual clinical deployment.

### 2.2 Voxel-wise multimodal registration and 3D panoramic segmentation

Multimodal image preprocessing and target volume delineation utilized the open-source platform 3D Slicer. Original images underwent rigorous image-level normalization before target delineation. For PET images, histogram matching calibrated SUV distribution across varying scanners, correcting baseline offsets due to detector sensitivity differences. For CE-MRI images, N4 bias field correction addressed magnetic field inhomogeneity, followed by Z-score transformation to standardize signal intensity. To ensure precise voxel-to-voxel spatial alignment between different dynamic CE-MRI phases prior to calculating temporal changes, a 3D B-spline deformable registration algorithm (utilizing mutual information as the similarity metric) aligned the arterial phase to the portal venous phase. This preprocessing enabled the adaptive segmentation algorithm, defined as “liver background mean SUV + 2 standard deviations,” to delineate metabolically active regions accurately within a unified physical framework, minimizing scanner noise interference on the binary fractal matrix.

Post-normalization, PET metabolic maps and CE-MRI images were spatially resampled to a uniform 1×1×1 mm³ 3D resolution. Images exhibiting severe respiratory motion artifacts were excluded. A mutual information-based 3D elastic registration algorithm subsequently aligned metabolically active regions with microvascular perfusion regions into a shared physical coordinate system (11). Radiologists visually verified liver contours and major internal vessel landmarks post-registration, calculating the Dice similarity coefficient for target lesions to confirm cross-modality alignment reliability.

To assess radiomic feature extraction robustness, two senior radiologists—blinded to pathological and clinical outcomes—independently delineated images from 50 randomly selected patients. This involved semi-automatic 3D whole-tumor volume of interest (VOI) segmentation, uniformly expanded by 3 mm outward to encompass the invasive margin at the “tumor-liver interface”. One radiologist blindly re-delineated the same images after two weeks to evaluate intra-observer consistency. In the subsequent feature selection process, only fractal features demonstrating intra- and inter-observer correlation coefficients (ICC) strictly > 0.80 were retained for machine learning modeling, eliminating measurement noise from subjective boundary delineation. Specifically, the final macroscopic fractal probes demonstrated excellent reproducibility: the intra-observer and inter-observer ICCs for the PET metabolic fractal dimension (*D_f_*) were 0.94 and 0.91, respectively, while the ICCs for the MRI vascular fractal acceleration (*A_fd_*) were 0.92 and 0.89, respectively.

For patients presenting with multiple intrahepatic metastases (cohort median = 3 lesions), we strictly avoided computing fractal dimensions across a disjointed global mask, as the box-counting dimension is mathematically invalid when applied to scattered, disconnected structures. Instead, an independent lesion-level analysis followed by patient-level aggregation was employed. Adhering to RECIST 1.1 guidelines, up to three dominant measurable target lesions (prioritizing the largest diameters and clear imaging boundaries) were selected per patient. The FRAC-V module computed the macroscopic fractal features (*A_fd_* and *D_f_*) for each target lesion independently.

To construct a representative patient-level parameter for downstream machine learning modeling and progression-free survival (PFS) analysis, a volume-weighted average of the fractal metrics from the selected target lesions was calculated. Let *V_i_* represent the 3D voxel volume of the *i*-th target lesion, and *F_i_* represent its corresponding fractal feature. The patient-level aggregated feature (*F_patient_*) was defined as:


$$F_{patient}=\frac{\sum \left(F_{i}\cdot V_{i}\right)}{\sum V_{i}}$$

This volume-weighted aggregation strategy accurately translates lesion-level spatial heterogeneity into a patient-level biophysical burden, ensuring robust input for the IRJS model.

### 2.3 Quantification of TIME barriers via FRAC-V

A custom FRAC-V module extracted 3D nonlinear fractal dynamics features from the complete tumor volume:

#### 2.3.1 Mathematical formulation and numerical procedures via FRAC-V

To quantify the macroscopic spatial chaos of the tumor immune microenvironment, we utilized a custom-developed radiomics engine, the FRAC-V suite, integrated as a module within the 3D Slicer platform. This allowed for the automated extraction of 3D nonlinear fractal dynamics based on rigorous mathematical formalisms.

The spatial fractal dimension for each imaging modality was computed utilizing a 3D finite-resolution box-counting algorithm. The binarized 3D region of interest (ROI) was superimposed with a grid of cubic boxes of side length *ϵ*. The number of boxes containing at least one metabolically active or vascular-enhanced voxel, N(ϵ), was counted. The box edge sizes were logarithmically scaled, $\epsilon \in \left\{2^{1},2^{2},2^{3},2^{4},2^{5},2^{6}\right\}$ voxels. The static fractal dimension (*D_f_*) was defined mathematically by the finite-resolution power law:


$$D_{f}=\underset{\epsilon \to 0}{\lim}\frac{\log N(\epsilon )}{\log(1/\epsilon )}$$

In practice, *D_f_* was estimated via Ordinary Least Squares (OLS) linear regression of logN(ϵ) against log(1/ϵ). To estimate mathematical uncertainty and ensure geometric validity, Goodness-of-Fit (*R*^2^) of the regression line was calculated for each lesion. Only features from regressions achieving an *R*^2^ ≥ 0.95 were retained for subsequent machine learning, guaranteeing that the target structures exhibited true fractal scaling behavior rather than random voxel noise.

#### 2.3.2 Metabolic fractal dimension (*D_f_*)

The spatial heterogeneity of the metabolic barrier was estimated using a 3D box-counting algorithm on the ^18^F-FDG PET images. Let the 3D bounding box of the tumor region of interest be partitioned into a grid of cubes with side length *r*. Let *N*(*r*) denote the minimum number of cubes required to cover all hyper-metabolic voxels. The metabolic fractal dimension, *D_f_*, is numerically approximated by the slope of the linear region in the log-log plot of *N*(*r*) versus 1/*r*:


$$D_{f}=\underset{r\to 0}{\lim}\frac{\log N\left(r\right)}{\log\left(1/r\right)}$$

In our FRAC-V implementation, box-edge sizes were logarithmically scaled from 2 to 64 voxels (2^1^ to 2^6^). A higher *D_f_* mathematically represents a more space-filling, chaotic glycolytic distribution, serving as our quantitative surrogate for metabolic exhaustion.

#### 2.3.3 Vascular fractal acceleration (*A_fd_*)

To capture the temporal perfusion kinetics of abnormal angiogenesis (the physical barrier), we computed the derivative of the vascular spatial complexity across dynamic contrast-enhanced MRI (CE-MRI) phases. The true vascular fractal acceleration *A_fd_* incorporates the unenhanced baseline phase (*t_unenh_* = 0 s) alongside the arterial (*t_art_* = 30 s) and portal venous (*t_pv_* = 70 s) phases to capture the ‘rate of change of the rate of change’ in spatial complexity. Let *D_f,art_* represent the vascular fractal dimension extracted during the arterial phase (*t_art_* = 30 seconds), and *D_f_*_,pv_ represent the dimension during the portal venous phase (*t_pv_* = 70 seconds), both calculated using the 3D box-counting method described above.


$$V_{in}=\frac{D_{f,art}-D_{f,unenh}}{t_{art}-t_{unenh}},V_{trans}=\frac{D_{f,pv}-D_{f,art}}{t_{pv}-t_{art}}$$

$$A_{fd}=\kappa \frac{V_{trans}-V_{in}}{\Delta t_{mean}}$$

To render the numerical values clinically intuitive, a scaling multiplier (*κ* = 100) was embedded in the final formulation, Δ*t_mean_* represents the mean of time interval.

#### 2.3.4 Quantification of the physical vascular barrier

To quantify the physical barrier, the vascular fractal acceleration (*A_fd_*) of contrast agent penetration across CE-MRI phases was extracted. Following inter-phase registration and adaptive intensity normalization to isolate hyper-enhancing vascular voxels, the spatial fractal dimension for each individual phase was computed using a 3D box-counting algorithm with box-edge sizes logarithmically scaled from 2 to 64 voxels. *A_fd_* was mathematically defined as the slope of the fractal dimension change from the arterial phase (30 seconds post-injection) to the portal venous phase (70 seconds post-injection), this genuine second-order parameter specifically isolates the rapid contrast pooling and tortuosity unique to chaotic tumor neovasculature. By distinguishing immature microvessels from normal hepatic parenchyma, high *A_fd_* values numerically quantify rapid, chaotic contrast hyper-leakage. Consequently, this metric functions purely as a non-invasive biophysical estimator mapping to the dense fibrovascular stroma—serving as a macroscopic quantifier of the “physical barrier” impeding CD8+ T cell infiltration, rather than a direct mechanistic mediator of resistance.

- Quantification of the metabolic exhaustion barrier: A multi-scale 3D box-counting method extracted the 3D metabolic fractal dimension (*D_f_*) from PET images. Prior to calculation, an adaptive threshold (individual liver background mean SUV + 2 standard deviations) converted PET images into a binary metabolically active matrix. The reference region of interest (ROI) for liver background SUV was defined as a 3 cm diameter spherical region within a lesion-free area of the right lobe. Extreme spatial heterogeneity in *D_f_* mapped the intense glycolytic competition between tumor cells and macrophages, quantifying the “metabolic barrier” driving T cell exhaustion.

To ensure that the calculated metabolic fractal dimension (*D_f_*) reflects genuine biological heterogeneity rather than interpolation artifacts or thresholding bias, a comprehensive numerical validation was performed. The robustness of *D_f_* to voxel resampling was verified by confirming a high Intraclass Correlation Coefficient (ICC > 0.90) between features extracted from the native PET resolution and the 1×1×1 mm³ interpolated grid. The strict inclusion threshold of regression *R*^2^ > 0.95 further guaranteed scale-invariance. A threshold sensitivity analysis confirmed that varying the PET binarization cutoff (e.g., Liver Background Mean + 3 SD, or fixed 40% SUVmax) did not significantly alter the cohort’s spatial heterogeneity hierarchy (Spearman *R* > 0.85), validating the mathematical stability of the chosen parameters.

- Harmonization of residual batch effects: Following the extraction of raw macroscopic fractal features (*A_fd_* and *D_f_*), the ComBat harmonization algorithm utilizing an empirical Bayesian framework was applied to address minor residual batch effects stemming from hardware aging or undisclosed reconstruction algorithm updates over the 5-year study. To prevent data leakage and strictly preserve the integrity of the independent temporal validation, the ComBat model was fitted *exclusively* on the 2019–2024 training cohort. The model utilized the scanner model as the batch variable and preserved variations related to the training cohort’s pathophysiological covariates (histological origin and immune phenotype). Once the empirical Bayes shift and scale parameters were estimated from the training data, these parameters were locked. This frozen harmonization model was then applied unaltered to the 2025 temporal validation cohort. The validation cohort’s labels and features were strictly excluded from the parameter estimation process, ensuring zero data leakage.

### 2.4 Digital pathology mapping of TIME

Digital quantification of whole-slide images (WSI) from specimens constructed a closed-loop biological evidence chain. Region-level spatial co-registration utilized gross specimen photography and 3D contour reconstruction of preoperative images. This ensured that the core and marginal areas of the pathological samples accurately matched the corresponding imaging-extracted regions of interest (12).

All IHC staining was executed on an automated immunohistochemistry staining platform (e.g., BenchMark ULTRA, Roche Diagnostic) following standardized procedures to ensure reproducibility. **Supplementary Table 2** details all technical parameters for core targets, including primary antibody clone numbers, suppliers, optimal dilution ratios, and antigen retrieval conditions. Based on this standardized staining, the absolute density or percentage positivity of specific IHC markers was evaluated:

- Vascular-stromal physical barrier: Quantification of microvascular density (CD31^+^) and smooth muscle actin (α-SMA) cross-validated the abnormal angiogenesis and dense stroma associated with high spatial exclusion.
- Metabolic exhaustion barrier: Quantification of the CD163^+^ cell population served as a surrogate for an immunosuppressive tumor-associated macrophage phenotype (13). This was combined with the quantification of abnormal GLUT-1 and PD-L1 expression to validate the biochemical competition and immune-tolerant microecology resulting from high metabolic heterogeneity.
- Common hypoxic microecology and spatial distribution: Hypoxia-inducible factor (HIF-1α) quantification assessed extreme microenvironmental stress. The absolute infiltration density of CD8^+^ T cells was systematically quantified in the tumor core (TC) and the invasive margin (IM, defined as 500 μm on either side of the tumor-liver interface). The margin-to-core entrapment ratio (IM/TC ratio) was calculated as the pathological gold standard for confirming spatial exclusion.

### 2.5 Comprehensive quantification of peripheral platelet-poor plasma cytokines

Fasting morning venous blood (EDTA-K2 anticoagulation) was collected before any systemic treatment and immediately placed on ice. Platelet-poor plasma (PPP) was prepared via dual-centrifugation at 4°C: an initial centrifugation at 1,500 × g for 15 minutes, followed by transferring the supernatant to a new tube for a second centrifugation at 10,000 × g for 10 minutes. Purified PPP was immediately aliquoted and frozen at -80°C to avoid repeated freeze-thaw cycles.

High-sensitivity ELISA kits (R&D Systems, USA) were used to blindly measure plasma concentrations of IFN-γ, TGF-β, and IL-10. TGF-β samples underwent routine acid-activation before measurement. All assays were performed in duplicate independent runs, with intra- and inter-assay coefficients of variation (CV) strictly controlled at<10%.

### 2.6 TIME-anchored machine learning modeling and confounder isolation

To avoid “shortcut learning” where the algorithm might simply classify baseline histological differences (CRLM vs. SCC) rather than capturing a universal spatial immune signature, the machine learning pipeline was rigorously decoupled from tumor origin. First, to isolate histological confounders, the macroscopic fractal probes (*A_fd_* and *D_f_*) were Z-score normalized strictly within their respective histological subgroups. This ensured that the spatial chaos of a tumor was evaluated relative only to other tumors of the identical histological origin. Subsequently, an extreme gradient boosting (XGBoost) classifier was constructed in the training cohort. The explicit ground-truth prediction target was defined by digital pathology as the “High TIME Barrier” phenotype. This phenotype was mathematically defined as having both restricted effector infiltration (core CD8^+^ density below the cohort median) and low immune target expression (PD-L1 TPS below the median).

The reliance on cohort-specific medians, rather than absolute clinical thresholds (e.g., PD-L1 TPS ≥ 1%), was driven by the dual-histology study design. Because CRLM and SCC exhibit intrinsically different baseline immunogenicities, applying a single absolute cutoff would disproportionately skew the ‘High TIME Barrier’ classification toward the less immunogenic CRLM subgroup, thereby reintroducing histological bias. The median-based approach facilitates the consistent identification of severe immune-excluded and microenvironmentally suppressed states across the cohort, irrespective of primary tumor origin.

Hyperparameter optimization involved 10-fold cross-validation combined with an exhaustive grid search strategy (detailed in **Supplementary Table 3**). The SHAP interpretative framework was applied to quantify how subgroup-normalized fractal parameters facilitate the prediction of this cold, immune-excluded state.

To justify the algorithmic selection, the predictive performance of XGBoost was benchmarked against five alternative machine learning architectures: Logistic Regression (LR), Support Vector Machine (SVM), Random Forest (RF), LightGBM, and a Multi-Layer Perceptron (MLP) neural network. All models were trained using identical 10-fold cross-validation protocols on the normalized feature set.

To ensure computational reproducibility, the complete hyperparameter search space, cross-validation protocols, and software dependencies (e.g., Scikit-learn v1.0.2, XGBoost v1.5.0) are meticulously detailed in the **Supplementary Methods**.

### 2.7 Construction of the immune-radiomics joint score and independent temporal validation

The individualized continuous prediction probability (ranging from 0 to 1) of harboring a “High TIME Barrier” phenotype generated by the XGBoost model was defined as the immune-radiomics joint score (IRJS). Adhering to standard chronological validation principles, the optimal IRJS cut-off value for distinguishing high- versus low-microenvironmental barrier risk was determined in the 2019–2024 training cohort by maximizing the Youden index (optimal cut-off IRJS = 0.64). This fixed threshold was then applied unaltered to the independent 2025 temporal validation cohort. A receiver operating characteristic (ROC) curve evaluated the model’s generalizable diagnostic accuracy.

### 2.8 Early dynamic imaging follow-up and sensitivity analysis cohort

Routine clinical follow-up for advanced liver metastases typically occurs 6–8 weeks post-diagnosis. To explore early TIME remodeling, a dynamic assessment subcohort of 80 patients receiving immunotherapy-based combination therapy was retrospectively screened. Because these ultra-early week 3 scans were prompted by specific clinical indications rather than routine scheduling, this subcohort is inherently susceptible to selection bias. Therefore, all survival analyses regarding week 3 dynamic fractal drift (Δ*A_fd_*) are strictly designated as exploratory. These scans were prompted by specific clinical indications (e.g., suspected pseudoprogression, early toxicity monitoring) or participation in an exploratory efficacy assessment protocol. To mitigate selection bias and validate the generalizability of these dynamic indicators in standard clinical practice, routine CE-MRI data from the standard clinical evaluation time point (weeks 6–8) were also collected from these patients for a parallel sensitivity analysis.

### 2.9 Extraction of dynamic fractal features (Δ*A_fd_*) and barrier tracking

The FRAC-V module extracted the 3D vascular fractal acceleration (*A_fd_*) from target lesion CE-MRIs at each follow-up point. Longitudinal fractal drift, such as Δ*A_fd_* at week 3 relative to baseline, served as a non-invasive dynamic indicator to assess tumor microvascular topological evolution and physical barrier remodeling (14). A significant negative drift (decrease) in Δ*A_fd_* functioned as an early surrogate marker of physical barrier disruption. The threshold defining a ‘significant negative drift’ (Δ*A_fd_*< −0.20) was strictly data-driven, determined by maximally selected rank statistics to maximize the log-rank separation for PFS within the dynamic subcohort. To confirm the robustness of this cut-off against landmark analysis sensitivity, a continuous sensitivity analysis was performed, verifying that therapeutic benefit remained consistent across adjacent threshold variations (-0.15 to -0.25). The complete evolution curve tracked the vascular normalization window and subsequent secondary barrier closure.

### 2.10 Clinical survival endpoints and multivariable analysis

Progression-free survival (PFS) was the primary endpoint, defined as the time from ICI therapy initiation to all-cause death or confirmed disease progression per iRECIST criteria. For patients showing initial imaging progression (iUPD), confirmation (iCPD) was required 4–8 weeks later; the PFS event date was recorded as the initial iUPD time point.

Kaplan-Meier survival curves were plotted. A multivariable Cox proportional hazards regression model controlled for clinical confounding bias, adjusting for covariates including baseline tumor burden, histological subtype, mismatch repair/microsatellite instability (MMR/MSI) status, and systemic treatment regimen (ICI monotherapy vs. combination therapy). The model independently assessed the predictive value of the continuous baseline IRJS (analyzed per 0.1-unit increment) and early dynamic Δ*A_fd_* for primary ICI resistance and long-term benefit.

Median follow-up time was calculated utilizing the reverse Kaplan-Meier method. Prior to executing the multivariable Cox proportional hazards regression, the proportional hazards (PH) assumption was rigorously verified for all variables using scaled Schoenfeld residuals. Both global and individual covariate statistical tests were performed to ensure that hazard ratios did not significantly vary over time.

### 2.11 Sensitivity analysis for histological confounding

To confirm that this newly defined score (IRJS) successfully decoupled the TIME signature from histological bias, a rigorous two-step downstream sensitivity validation was performed. First, to evaluate prognostic independence, multivariable Cox proportional hazards regressions utilizing the continuous IRJS were executed separately within the CRLM and SCC immunotherapy sub-cohorts. This step was designed to confirm that the IRJS maintained its dominant predictive value for progression-free survival (PFS) irrespective of the primary tissue histology, thereby establishing the “physical-metabolic” spatial barriers as a universal prognostic character. Second, independent XGBoost model retraining with 10-fold cross-validation was conducted within the pure CRLM (n=195) and pure SCC (n=277) cohorts, deliberately omitting primary tissue histology labels. Re-extracting SHAP contribution matrices for these single-cancer models verified that the fractal spatial metrics Δ*A_fd_* and *D_f_*) retained their dominant prognostic driving weights across different tumor origins.

### 2.12 Statistical analysis

Continuous variables were reported as median (interquartile range) or mean ± standard deviation, with group comparisons performed using the Mann-Whitney *U* test or Student’s *t*-test. Regarding missing data, a strict complete-case analysis approach was adopted for all core multimodal variables. As detailed in the patient selection flowchart, patients lacking baseline ^18^F-FDG PET/CT, CE-MRI, complete immunohistochemical specimens, or definitive survival follow-up were rigorously excluded. For minor missingness in routine baseline clinical laboratory parameters (missing rate< 5%), missing values were addressed using simple median imputation for continuous variables prior to machine learning and multivariable Cox modeling. Categorical variables were evaluated using the chi-square test or Fisher’s exact test. Spearman’s rank correlation analysis assessed cross-scale correlations between multi-omic features, micro-histochemical, and serological indicators. To control for false positives in multiple testing, correlation matrix *P* values were adjusted via the Benjamini-Hochberg false discovery rate (FDR) method.

Univariate survival analysis used the Kaplan-Meier method, and between-group significance was tested with the log-rank test. Multivariable Cox proportional hazards regression identified independent prognostic factors, providing hazard ratios (HR) and 95% confidence intervals (CI). Area under the ROC curve (AUC) and decision curve analysis (DCA) evaluated prediction model discriminative ability and clinical utility, respectively. To evaluate the accuracy of the predicted probabilities, model calibration was visually assessed via calibration plots. Quantitative calibration performance was evaluated using the Brier score and the Hosmer-Lemeshow (H-L) goodness-of-fit test, where a *P*-value > 0.05 indicates a well-calibrated model. Python (version 3.8.10) and R (version 4.1.2) were used for all statistical analyses and machine learning modeling. All hypothesis tests were two-sided, with *P* < 0.05 deemed statistically significant.

## 3 Results

### 3.1 Baseline cohort characteristics and bipolar macroscopic fractal dynamics

To mitigate data leakage bias, we employed a strict chronological split for the 472 enrolled treatment-naive patients with liver metastases. A training set (n = 400) from June 2019 to December 2024 and an independent validation set (n = 72) from 2025 were established. The entire cohort comprised 195 cases of colorectal cancer liver metastases (CRLM, representing the adenocarcinoma phenotype) and 277 cases of lung squamous cell carcinoma liver metastases (SCC, representing the squamous phenotype). **Table 1** details the baseline clinical, molecular pathological, radiomic, and immunological patient characteristics.

**Table 1:** Baseline demographic, clinical, radiomic, and immuno-pathological characteristics of the study cohort, stratified by tumor phenotype.

Characteristics	Total cohort (N = 472)	CRLM cohort (n = 195)	SCC cohort (n = 277)	P value
Demographics				
Age (years), mean ± SD	63.5 ± 9.8	62.8 ± 10.2	64.1 ± 9.4	0.165
Sex (Male/Female), n	312/160	115/80	197/80	< 0.001
ECOG Performance Status (0-1/2), n	385/87	162/33	223/54	0.542
Tumor burden & clinical markers				
Number of liver lesions, median (IQR)	3 (2–5)	3 (1–5)	3 (2–6)	0.384
Max lesion diameter (cm), mean ± SD	4.5 ± 1.8	4.6 ± 1.9	4.4 ± 1.7	0.256
Baseline Serum LDH (U/L), median (IQR)	245 (185–310)	238 (175–305)	252 (190–315)	0.125
Neutrophil-to-Lymphocyte Ratio (NLR) ≥ 4.0, n (%)	215 (45.6%)	85 (43.6%)	130 (46.9%)	0.485
Macroscopic fractal dynamics (non-invasive probes)				
PET Metabolic Fractal Dimension (Df), mean ± SD	2.50 ± 0.25	2.70 ± 0.18	2.36 ± 0.22	< 0.001
MRI Vascular Fractal Acceleration (Afd), mean ± SD	1.73 ± 0.52	1.19 ± 0.35	2.12 ± 0.41	< 0.001
Microscopic time phenotypes (local)				
CD8+ Margin-to-Core Ratio, median (IQR)	2.8 (1.5–4.2)	1.6 (1.1–2.2)	4.5 (3.2–6.8)	< 0.001
CD163+ Macrophages (TAMs) (%), mean ± SD	69.4 ± 11.2	75.2 ± 8.5	65.4 ± 11.5	< 0.001
HIF-1α High Expression, n (%)	242 (51.3%)	135 (69.2%)	107 (38.6%)	< 0.001
CD31+ Microvessel Density (MVD), mean ± SD	45.2 ± 12.5	38.5 ± 10.2	49.8 ± 11.8	< 0.001
Systemic immune exhaustion (peripheral)				
CD4+/CD8+ Ratio, mean ± SD	1.55 ± 0.48	1.62 ± 0.45	1.50 ± 0.51	0.012
Serum TGF-β (pg/mL), median (IQR)	25.4 (15.2–35.8)	32.5 (22.4–42.5)	20.4 (12.5–28.5)	< 0.001

SD, standard deviation; IQR, interquartile range; ECOG, Eastern Cooperative Oncology Group; LDH, lactate dehydrogenase; TAMs, tumor-associated macrophages; HIF-1α, hypoxia-inducible factor 1α.

Statistical comparisons between the CRLM and SCC cohorts were performed using the Student’s *t*-test for normally distributed continuous variables, the Mann-Whitney *U* test for non-normally distributed variables, and the Pearson *χ^2^* test for categorical variables. Bold P values indicate statistical significance (*P* < 0.05).

To prevent immune resistance interference from intrinsic genotypes, the mismatch repair/microsatellite instability (MMR/MSI) status within the CRLM cohort was quantified. The majority of the CRLM subgroup demonstrated a microsatellite stable/mismatch repair-proficient profile (MSS/pMMR, 94.8%, 185/195), while a small fraction presented as microsatellite instability-high/mismatch repair-deficient (MSI-H/dMMR, 5.2%, 10/195). This baseline propensity toward a “cold tumor” confirmed that the observed ICI treatment failures were primarily facilitated by “microenvironmental physical/metabolic spatial barriers” rather than intrinsic genetic resistance. Comparisons between the historical training set and the 2025 independent validation set characterized no statistically significant differences in age, sex, baseline tumor burden, or the distribution of systemic treatment regimens (all *P* > 0.05, detailed in **Supplementary Table 1**), ruling out clinical confounding from chronological drift.

**Figure 1:**
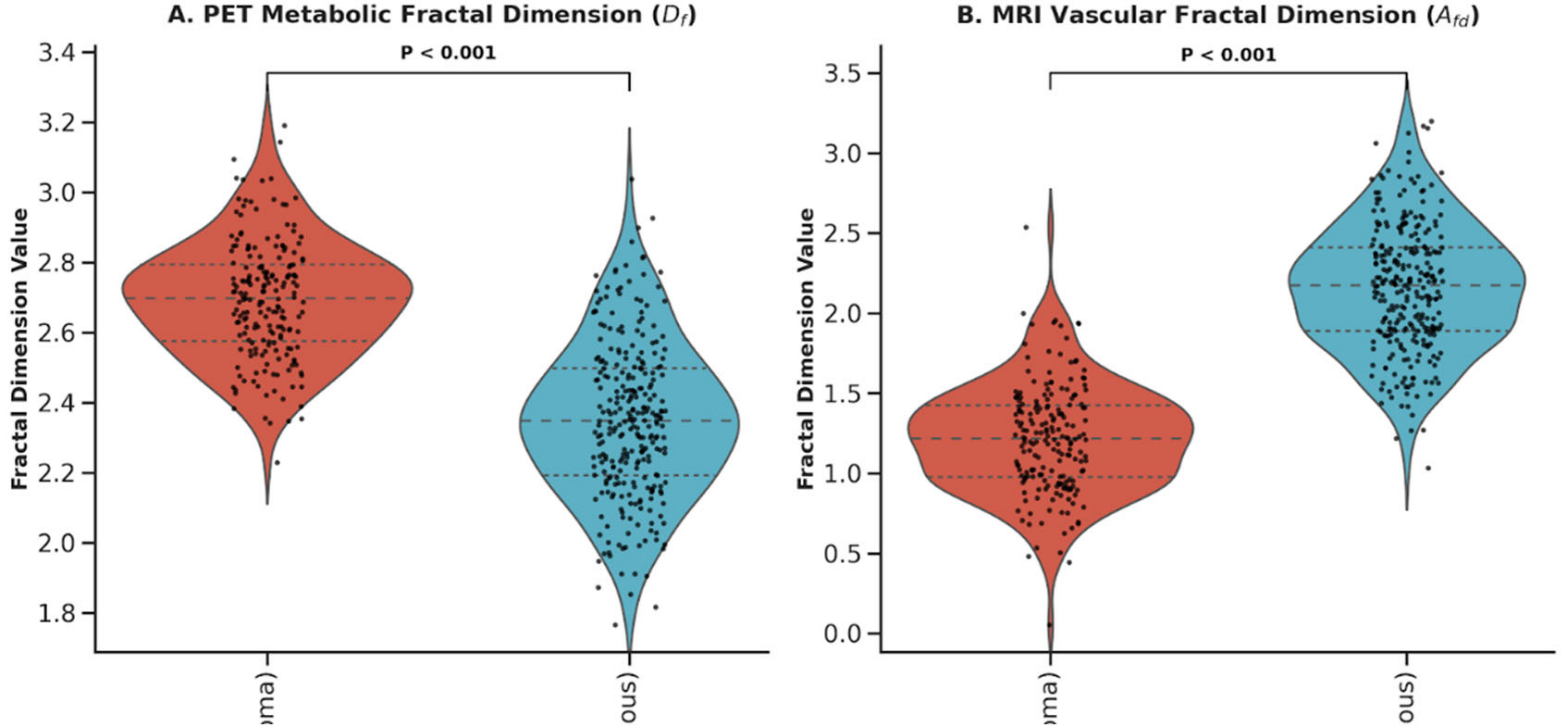
Divergent macroscopic fractal dynamics characterize distinct physical and metabolic barrier phenotypes in CRLM and SCC liver metastases. **(A)** Distribution of the PET metabolic fractal dimension (*D_f_*) across the two cohorts. Colorectal liver metastases (CRLM, adenocarcinoma phenotype, red) exhibit a significantly higher *D_f_* compared to squamous cell carcinoma (SCC) metastases (blue), indicating intense spatial glycolytic chaos and a significant metabolic barrier. **(B)** Distribution of the MRI vascular fractal dimension (*A_fd_*). Conversely, SCC metastases demonstrate a dramatically elevated *A_fd_*, reflecting highly tortuous and chaotic neovasculature that constitutes a severe physical barrier to immune infiltration. Violin plots show the probability density of the data at different values, with interior dashed lines representing the median and interquartile ranges (IQR). Black dots represent individual patient data points (CRLM, n = 195; SCC, n = 277). Statistical significance was determined using the Mann-Whitney *U* test (*P* < 0.001).

Regarding macroscopic tumor burden, the CRLM and SCC cohorts showed no significant differences in the number of liver metastases (median 3 in both, *P* = 0.384) or maximum diameter (4.6 cm *vs.* 4.4 cm, *P* = 0.256). However, macroscopic fractal dynamics extracted from non-invasive imaging indicated distinct spatial conformations (**Figure 1**). The CRLM cohort displayed an elevated PET metabolic fractal dimension (*D_f_* = 2.70 ± 0.18, *P* < 0.001), indicating high intratumoral glycolytic spatial heterogeneity. Conversely, the SCC cohort presented an abnormally increased MRI vascular fractal acceleration (*A_fd_* = 2.12 ± 0.41, *P* < 0.001). This divergence suggests that the microenvironmental evolution of adenocarcinoma and squamous cell carcinoma liver metastases depends heavily on extreme metabolic and physical space barriers, respectively.

### 3.2 Cross-scale mapping: macroscopic fractal features characterize the “dual microenvironmental barriers” and systemic immune cascade

**Figure 2:**
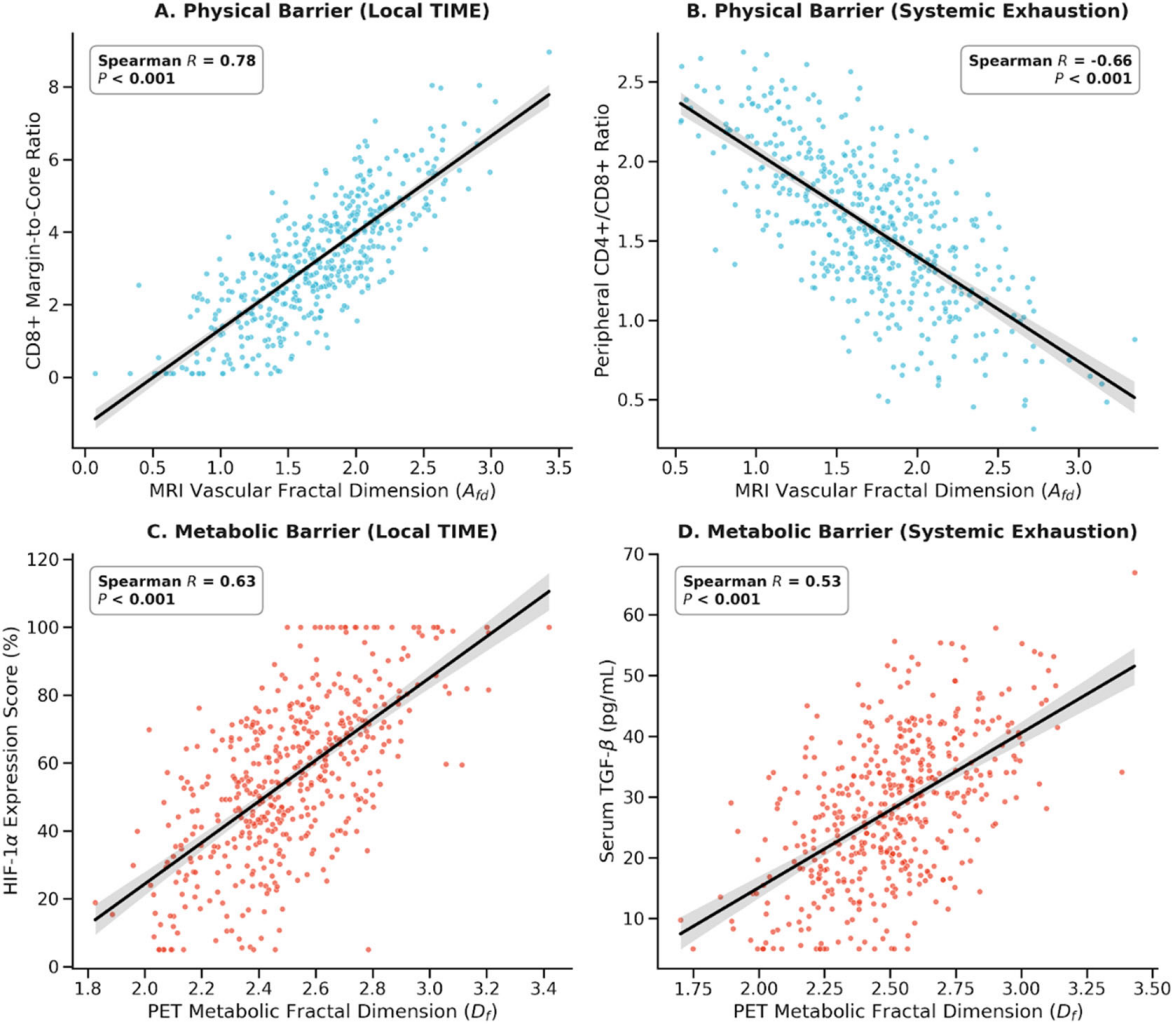
Cross-scale mutual validation mapping macroscopic fractal dynamics to local and systemic immunological phenotype. **(A)** The MRI vascular fractal dimension (*A_fd_*) strongly correlates with the CD8^+^ Margin-to-Core Ratio (Spearman *R* = 0.78, *P* < 0.001), confirming its validity as a macroscopic surrogate for the physical barrier mediating spatial immune exclusion. **(B)** Elevated *A_fd_* is significantly associated with an inverted peripheral CD4^+^/CD8^+^ ratio (*R* = -0.66, *P* < 0.001), extending the concept of the physical barrier to systemic cellular exhaustion. **(C)** The PET metabolic fractal dimension (*D_f_*) is highly correlated with the local HIF-1α expression score (*R* = 0.63, *P* < 0.001), validating its role as an *in vivo* probe for the metabolic barrier induced by intense spatial glycolysis and hypoxia. **(D)** High *D_f_* corresponds to a systemic surge in immunosuppressive serum TGF-β (*R* = 0.53, *P* < 0.001), further linking local metabolic chaos to global immune failure. Solid black lines represent linear regression fits, with gray shaded areas indicating 95% confidence intervals. Spearman’s rank correlation (*R*) was used to evaluate monotonic associations.

**Figure 3:**
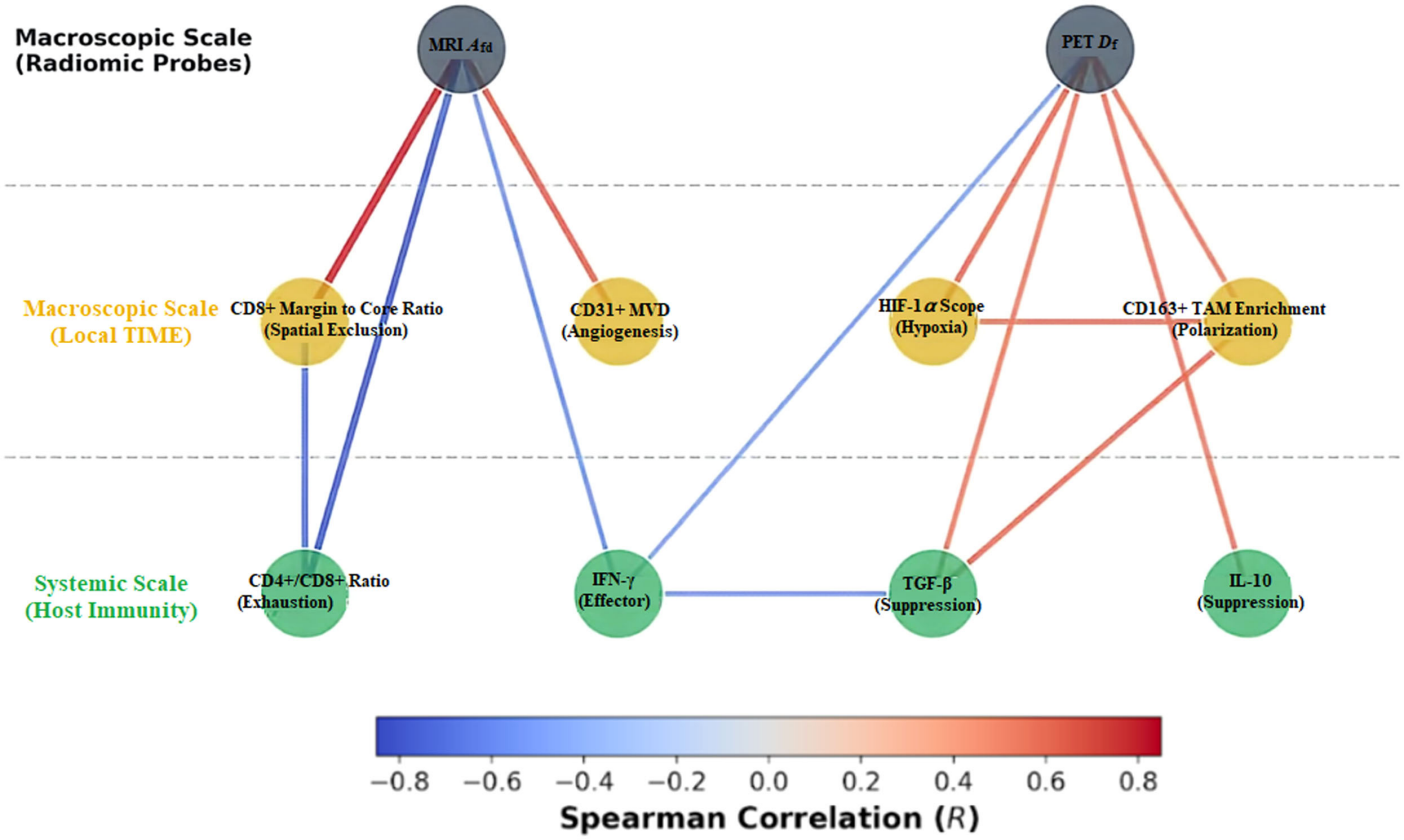
Cross-scale interactive network of the dual-barrier microenvironment in liver metastases. This tripartite network diagram illustrates the significant correlations between macroscopic radiomic probes (top tier), the local tumor immune microenvironment (TIME, middle tier), and systemic host immunity (bottom tier). The edges represent Spearman’s rank correlations (*R*), with line thickness proportional to the absolute correlation strength. Warm colors (red) indicate positive correlations, whereas cool colors (blue) indicate negative correlations. The network characterizes two distinct mechanistic axes: (1) The Physical Axis (left): The MRI vascular fractal dimension (*A_fd_*) is strongly associated with local spatial exclusion (CD8^+^ Margin/Core Ratio) and systemic T-cell exhaustion (inverted CD4^+^/CD8^+^ ratio). (2) The Metabolic Axis (right): The PET metabolic fractal dimension (*D_f_*) correlates with local hypoxia (HIF-1α) and macrophage polarization (CD163^+^), culminating in a systemic surge of immunosuppressive cytokines (TGF-β, IL-10) and the depletion of effector IFN-γ. This structural mapping validates the Immuno-Radiomic Joint Score (IRJS) as a holistic surrogate for the dual-barrier TIME.

An in-depth cross-scale correlation analysis evaluated the biological plausibility of macroscopic fractal dynamics (*D_f_* and *A_fd_*) as “virtual biopsy” probes of the tumor immune microenvironment (TIME) (**Table 2**; **Figure 2**, **3**).

**Table 2:** Cross-scale mutual validation: Spearman correlation between macroscopic fractal dynamics and immunological phenotypes (N = 472).

Immunological biomarkers	PET metabolic fractal dimension (D_f_)		MRI vascular fractal dimension (A_fd_)	
	Spearman R	P value	Spearman R	P value
Local tumor immune microenvironment (TIME)				
CD8+ Margin-to-Core Ratio (Spatial Exclusion)	0.28	0.045	**0.78**	**< 0.001**
CD31+ Microvessel Density (MVD)	0.35	0.012	**0.65**	**< 0.001**
HIF-1α Expression Score (Hypoxia)	**0.63**	**< 0.001**	0.31	0.028
CD163+ Macrophages (TAMs) (%)	**0.54**	**< 0.001**	0.25	0.062
Systemic immune & cytokine profiling				
Serum TGF-β (pg/mL)	**0.53**	**< 0.001**	0.38	0.008
Serum IL-10 (pg/mL)	**0.57**	**< 0.001**	0.32	0.022
Serum IFN-γ (pg/mL)	**-0.48**	**< 0.001**	**-0.52**	**< 0.001**
Peripheral Blood CD4+/CD8+ Ratio	-0.42	0.004	**-0.66**	**< 0.001**
Neutrophil-to-Lymphocyte Ratio (NLR)	0.45	0.002	**0.68**	**< 0.001**

*D_f_*, metabolic fractal dimension; *A_fd_*, vascular fractal dimension; TAMs, tumor-associated macrophages; HIF-1α, hypoxia-inducible factor 1α.

Correlation analysis was performed using Spearman’s rank-order correlation coefficient (*R*) to capture monotonic, non-linear relationships across continuous scales. Bold text highlights strong correlations (|R| > 0.50, *P* < 0.001) that define the physical and metabolic barrier axes. Bold values indicate strong correlations (|R| > 0.50, P < 0.001) that define the physical and metabolic barrier axes.

#### 3.2.1 Physical barrier axis: “spatial exclusion” accompanied by combined vascular-stromal remodeling

Traditional radiomics often link morphological features solely to angiogenesis. This study, however, broadens that perspective. As detailed in **Table 2**, the MRI vascular fractal dimension (*A_fd_*) was significantly positively correlated with microvascular density (CD31^+^, *R* = 0.65, *P* < 0.001) and showed a strong covariation with the density of α-SMA^+^ cancer-associated fibroblasts (CAFs), an indicator of dense fibrous stroma (*R* = 0.76, *P* < 0.001). Scatter plot regression (**Figure 2**) confirmed a monotonic increase in the margin-to-core entrapment ratio of CD8^+^ T cells as *A_fd_* values rose (*R* = 0.78, *P* < 0.001). This demonstrates that, *in vivo*, a high vascular fractal feature identifies a deformed and fibrotic “fibrovascular niche.” High *A_fd_* was associated with severe CD8^+^ spatial exclusion, suggesting the dense physical matrix network restricts effector T cells to the invasive tumor margin, creating an “immune-excluded” state (15).

#### 3.2.2 Metabolic barrier axis: “biochemical immunosuppression” associated with spatial hypoxia

For the CRLM adenocarcinoma phenotype, immune resistance is largely facilitated by local metabolic ecosystem remodeling. Regression analysis established a correlation between the PET metabolic fractal dimension (*D_f_*) and the local HIF-1α expression score (*R* = 0.63, *P* < 0.001), demonstrating that macroscopic tracer spatial heterogeneity maps microscopic tissue hypoxia. Furthermore, *D_f_* correlated positively with elevated expression of the glucose transporter GLUT-1 and PD-L1 on tumor cells, confirming that glycolytic competition signals a hostile, immune-tolerant microecology. Elevated *D_f_* was also accompanied by increased infiltration of CD163**^+^** tumor-associated macrophages (TAMs) (*R* = 0.53, *P* < 0.001). This indicates that under extreme hypoxia and biochemical competition, microenvironmental metabolic reprogramming aligns with macrophage polarization toward a pro-tumor, suppressive phenotype, characterizing the metabolic barrier that inhibits T cell function.

#### 3.2.3 Cross-scale cascade and systemic anchor: systemic immune exhaustion is strongly associated with local barriers

To assess the link between the local microenvironment and systemic immune downregulation, we examined correlations between intratumoral cellular phenotype and peripheral serum factors. The density of CD163^+^ macrophages in the local tumor stroma showed a strong, positive correlation with the concentration of immunosuppressive TGF-β in peripheral serum (*R* = 0.68, *P* < 0.001). The network topology in **Figure 3** indicates a signaling cascade: local hypoxia (mapped by high *D_f_*) correlated with CD163^+^ macrophage enrichment. This polarization was accompanied by persistently elevated TGF-β in the peripheral systemic circulation, a significant downregulation of the anti-tumor cytokine IFN-γ (*R* = -0.48), and an inversion of the CD4^+^/CD8^+^ ratio.

This cross-scale evidence characterizes a strong association between local microenvironmental barriers and systemic immune exhaustion (16). While our cross-sectional design cannot establish causality, these findings indicate that liver metastases exhibiting extreme microenvironmental barriers are tightly linked to elevated systemic inhibitory signals, indicating a weakening of overall host immuno-fitness (17).

### 3.3 Construction of IRJS, temporal generalization validation, and dual-phenotype spatial immune principles

Integrating the subgroup-normalized *D_f_* and *A_fd_* in the training cohort, we constructed the immune-radiomics joint score (IRJS). By targeting the IHC-defined microenvironment, the IRJS effectively discriminated the ‘High TIME Barrier’ phenotype (AUC = 0.935) (**Table 3**; detailed confusion matrices are provided in **Supplementary Table 5**).

**Table 3:** Diagnostic performance for predicting the high TIME barrier phenotype in liver metastases: training vs. prospective temporal validation cohort.

Diagnostic models & probes	Training cohort (2019.6–2024.12, n = 400)				Temporal validation cohort (2025.1–2025.12, n = 72)			
	AUC (95% CI)	Sensitivity (%)	Specificity (%)	Accuracy (%)	AUC (95% CI)	Sensitivity (%)	Specificity (%)	Accuracy (%)
Clinical Baseline Model *	0.725 (0.685–0.765)	65.4	68.2	67.0	0.718 (0.642–0.794)	63.8	66.5	65.3
Single-modality probes								
PET Metabolic Fractal (Df)	0.835 (0.798–0.872)	78.5	76.4	77.5	0.820 (0.755–0.885)	75.0	78.6	76.4
MRI Vascular Fractal (Afd)	0.852 (0.815–0.889)	82.3	75.8	79.2	0.845 (0.782–0.908)	80.5	77.4	79.1
Immuno-radiomic joint score								
IRJS Model (Df + Afd)	0.935 (0.912–0.958) †	88.2	86.5	87.5	0.912 (0.865–0.959) ††	86.4	85.7	86.1

AUC, area under the receiver operating characteristic curve; CI, confidence interval; CRLM, colorectal liver metastases; SCC, squamous cell carcinoma; IRJS, Immuno-Radiomic Joint Score.

The Clinical Baseline Model incorporates standard non-imaging biomarkers, including serum Carcinoembryonic Antigen (CEA), Squamous Cell Carcinoma Antigen (SCC-Ag), and Lactate Dehydrogenase (LDH).

**†** *P<* 0.001 for DeLong’s test comparing the AUC of the IRJS model versus the Clinical Baseline Model (ΔAUC = 0.210, 95% CI: 0.155–0.265) and versus the best single-modality probe, MRI *A_fd_* (ΔAUC = 0.083, 95% CI: 0.045–0.121) in the Training Cohort.

**††** *P<* 0.001 for DeLong’s test comparing the AUC of the IRJS model versus the Clinical. Baseline Model (ΔAUC = 0.194, 95% CI: 0.095–0.293) and versus the single-modality probe MRI *A_fd_* (ΔAUC = 0.067, 95% CI: 0.015–0.119) in the Temporal Validation Cohort.

Note that the cutoff threshold applied to the validation cohort was strictly locked from the training cohort’s maximum Youden index, ensuring zero data leakage.

The corresponding confusion matrices detailing true/false positives and negatives for the training and temporal validation cohorts are available in **Supplementary Table 5**.

#### 3.3.1 High robustness validation across time sequences

**Figure 4:**
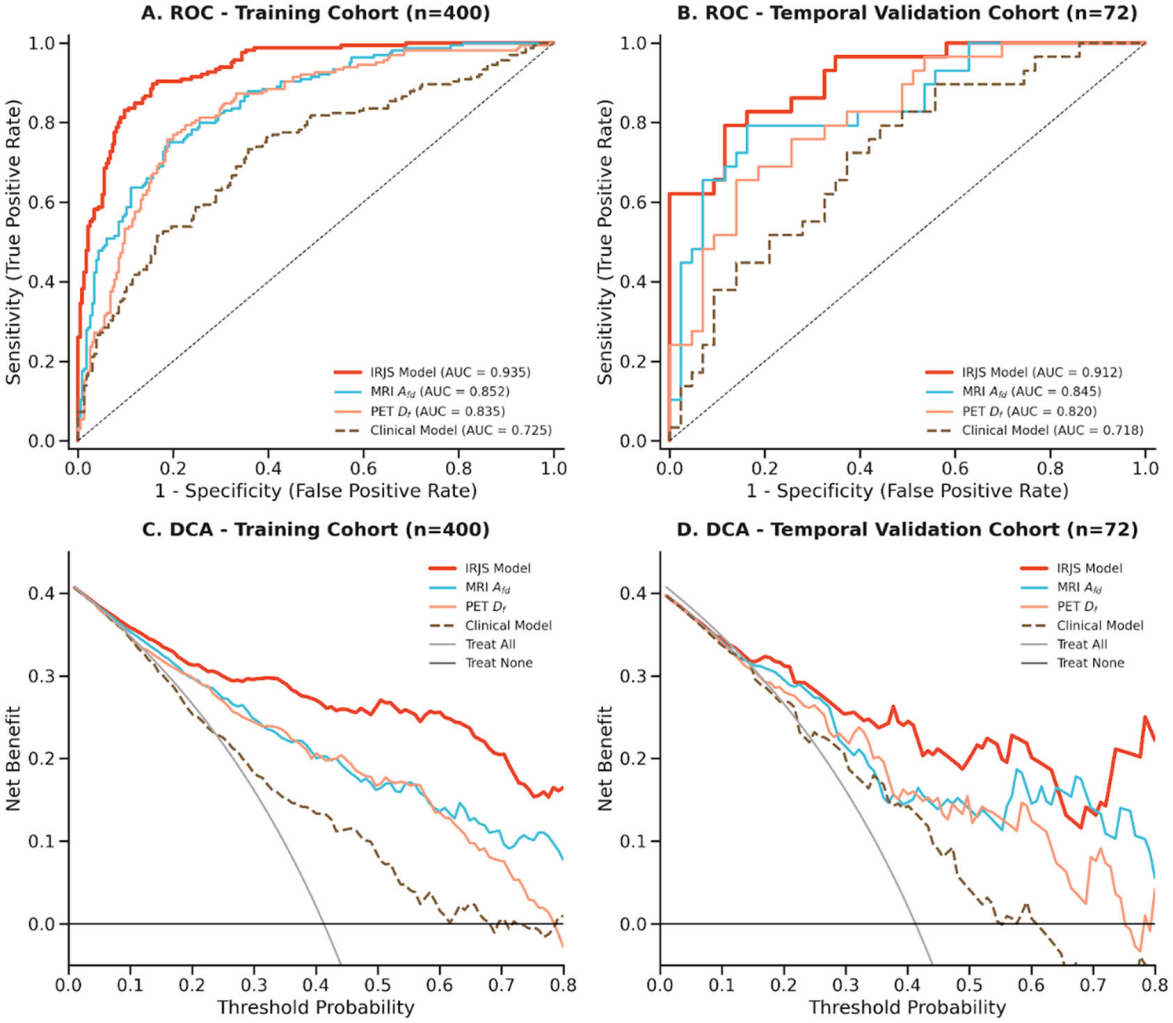
Diagnostic performance and clinical utility of the Immuno-Radiomic Joint Score (IRJS) for predicting the high TIME barrier phenotype in liver metastases. **(A)** Receiver operating characteristic (ROC) curves in the training cohort (2019–2024, n=400). The IRJS model (red, AUC = 0.935) significantly outperformed single-modality probes (*A_fd_* and *D_f_*) and the traditional clinical baseline model (dashed brown). **(B)** ROC curves in the independent prospective temporal validation cohort (2025, n=72). The IRJS model demonstrated robust generalizability with an AUC of 0.912, preventing overfitting and confirming its reliability across different timeframes. **(C, D)** Decision curve analysis (DCA) in the training and validation cohorts, respectively. The IRJS model consistently provided the highest net clinical benefit across a wide range of threshold probabilities (0.05 to 0.80) compared to alternative models and the default strategies of treating all or treating none, offering substantial value for Multidisciplinary Team (MDT) decision-making.

To mitigate overfitting risks and strictly adhere to standard temporal validation designs, the parameters from the 2019–2024 training cohort were locked. The IRJS maintained stable diagnostic performance in predicting the TIME phenotype in the independent 2025 temporal validation cohort with AUC = 0.912, as shown in **Figures 4A, B**. This robust temporal generalization confirms that the IRJS captures conserved ‘physical-metabolic spatial principles’ governing immune exclusion, independent of associating histology.

Beyond robust discrimination, evaluating model calibration is essential for clinical translation. Following *post-hoc* Platt scaling, the IRJS demonstrated excellent calibration in the training cohort (Brier score = 0.091; Hosmer-Lemeshow test *P* = 0.623), with predicted probabilities closely aligning with the 45-degree ideal line. Importantly, this robust calibration successfully generalized to the independent 2025 temporal validation cohort (Brier score = 0.128; Hosmer-Lemeshow test *P* = 0.415). These results confirm that the IRJS mitigates the extreme margin ‘overconfidence’ typical of tree-based ensembles and improves the ability for individual clinical risk assessment (**Supplementary Figure 4**). Decision curve analysis (DCA) demonstrated that the IRJS provides net clinical benefit across a wide range of threshold probabilities, supporting its use as an MDT decision-support tool, **Figures 4C, D**.

However, it must be acknowledged that because the temporal validation cohort was drawn from the same single institution, the patient demographics and imaging protocols were highly homogeneous with the training set (**Supplementary Table 1**). Consequently, the high AUC of 0.912 primarily reflects temporal stability against chronological drift, and true broad generalizability cannot be fully established without future external validation.

#### 3.3.2 Exclusion of histological confounding and establishment of cross-histology principles

Subgroup analyses were conducted to confirm the IRJS captures spatial microenvironments across cancer types, rather than acting as a histological classifier (**Supplementary Figure 1**). Within both the pure CRLM subgroup (HR = 2.45, *P* = 0.002) and the pure SCC subgroup (HR = 2.71, *P* = 0.005), a high IRJS score predicted a survival disadvantage. This indicates that in advanced metastasis, the systemic immunosuppressive pressure from spatial microenvironmental heterogeneity (quantified by macroscopic fractal dynamics) overrides cellular histology, establishing the “dual barrier” as a cross-histology spatial immune principle.

A comprehensive algorithmic benchmarking analysis (**Supplementary Table 4**) confirmed XGBoost as the optimal classifier for this dataset. XGBoost outperformed classical linear models (LR: AUC = 0.792; SVM: AUC = 0.815), alternative tree-based ensembles (RF: AUC = 0.895; LightGBM: AUC = 0.928), and a baseline deep learning model (MLP: AUC = 0.875, which exhibited slight overfitting). Consequently, XGBoost was selected as the final mathematical engine for the IRJS.

### 3.4 Interpretability analysis of the advanced machine learning model and identification of nonlinear thresholds

Game-theoretic SHAP values were utilized for global and local mechanistic analysis to address the “black box” nature of the extreme gradient boosting (XGBoost) algorithm and establish biological thresholds (18).

#### 3.4.1 Feature weights and global contribution excluding morphological confounding:

Following recursive feature elimination (RFE), the global SHAP summary plot indicated that the subgroup-normalized MRI vascular fractal acceleration (*A_fd_*) and PET metabolic fractal dimension (*D_f_*) were the primary features driving the model’s classification of the ‘High TIME Barrier’ (CD8^+^ low/PD-L1 low) phenotype. After neutralizing baseline histological variance prior to training, our results confirm that these macroscopic fractal parameters the biophysical spatial exclusion of the tumor, exceeding the marginal contributions of conventional clinicopathological indicators, such as tumor diameter, number of intrahepatic lesions, and patient age. This suggests the model captures the biophysical spatial essence of the tumor, rather than fitting the prognostic disadvantage from macroscopic tumor burden.

#### 3.4.2 Exploratory nonlinear thresholds and model-derived inflection point

SHAP local dependence plots identified the “nonlinear threshold” effects of macroscopic fractal dynamics on microenvironmental drivers. When *A_fd_* exceeded a specific threshold (e.g., *A_fd_* > 1.85), the corresponding SHAP prediction value increased exponentially. This maps to a critical transition where vascular-stromal remodeling corresponds to the physical barrier closing, sharply amplifying spatial exclusion risk. Similarly, when *D_f_* exceeded an inflection point near 2.55, the algorithm’s predicted probability of a ‘severe metabolic exhaustion’ phenotype increased significantly. These explainable AI findings provide exploratory, quantitative targets that may help hypothesize the timing of microenvironmental collapse, though they currently reflect algorithmic decision boundaries rather than independently validated biological bottlenecks.

The failure of the baseline logistic regression model to match XGBoost performance is explained by these highly non-linear threshold dynamics. For example, when *A_fd_* exceeded a specific threshold (*A_fd_* > 1.85), the corresponding SHAP prediction value increased exponentially, not linearly. This maps to a critical transition where vascular-stromal remodeling causes the physical barrier to close abruptly, sharply amplifying spatial exclusion risk. XGBoost’s ability to capture these threshold-dependent biological ‘bottleneck’ effects justifies its selection over simpler linear algorithms.

#### 3.4.3 Avoiding shortcut learning and validating CRLM and SCC feature dominance

Independent XGBoost retraining and SHAP sensitivity analysis within pure CRLM and SCC cohorts evaluated whether the model was confounded by “histological origin” (**Supplementary Figure 3**). Even when stripped of primary tumor histological labels, MRI vascular fractal acceleration (*A_fd_*) and PET metabolic fractal dimension (*D_f_*) remained the most important features. In the pure CRLM cohort, the positive SHAP contribution of high *D_f_* to the tolerant phenotype was 0.42; in the pure SCC cohort, the positive SHAP contribution of high *A_fd_* to the excluded phenotype was 0.47, exceeding tumor burden and other baseline characteristics. This sensitivity cross-validation establishes that the “physical-metabolic” dual spatial barriers represent an associating CRLM and SCC immunological principle.

### 3.5 Panoramic systemic immune profiling stratified by IRJS

**Figure 5:**
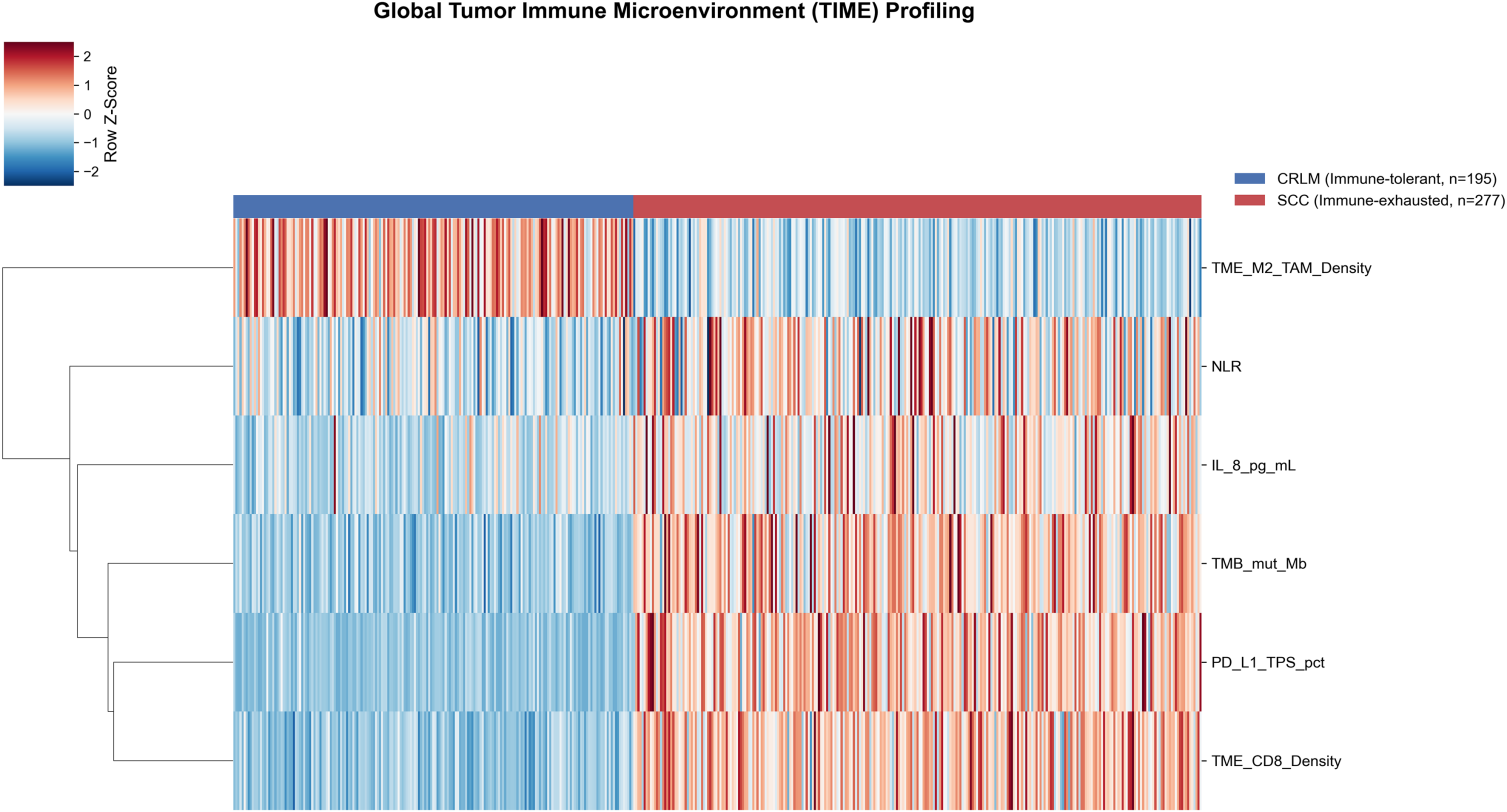
Comprehensive immuno-radiomic landscape mapping macroscopic fractal probes to local and systemic dual-barrier phenotypes. The heatmap illustrates the multi-scale biological alignment across 150 representative patients from the cohort, sorted from left to right by their ascending Immuno-Radiomic Joint Score (IRJS). The top annotation bars indicate the IRJS Risk stratification (Low vs. High Dual-Barrier) and the primary tumor phenotype (CRLM vs. SCC). As the macroscopic fractal dimensions (*A_fd_* and *D_f_*, top rows) intensify, a synchronized collapse of the immune landscape is observed: local physical exclusion (CD8^+^ Margin/Core Ratio, CD31^+^ MVD) and metabolic suppression (HIF-1α, CD163^+^) become severely upregulated (red zones). Concurrently, this local collapse triggers systemic immune exhaustion, evidenced by the depletion of serum IFN-γ and an inverted peripheral CD4^+^/CD8^+^ ratio (blue zones in the bottom rows), alongside a surge in immunosuppressive cytokines (TGF-β, IL-10). Expression values and fractal dimensions are Z-score normalized for optimal color contrast.

The entire study cohort (n = 472) was stratified into “low IRJS risk” and “high IRJS risk” groups using the optimal cut-off threshold from the training cohort (IRJS = 0.64), leading to the construction of a microenvironmental panoramic heatmap (**Figure 5**). As shown in **Table 4** and the heatmap, an increasing IRJS score correlated with comprehensive immunosuppressive remodeling. The high IRJS group exhibited high local hypoxic burden (67.8% with high HIF-1α expression) and dense effector cell spatial exclusion (median CD8^+^ margin-to-core ratio of 4.2). Peripheral blood indicators also showed exhaustion features: reduced levels of IFN-γ and increased concentrations of immunosuppressive cytokines like TGF-β and IL-10. This validated the association between highly heterogeneous liver metastases and host systemic immune dysfunction.

**Table 4:** Local and systemic immunological landscapes stratified by the Immuno-Radiomic Joint Score (IRJS).

Immunological biomarkers	Low IRJS risk (low dual-barrier, n = 236)	High IRJS risk (high dual-barrier, n = 236)	P value
Local tumor immune microenvironment(TIME)			
CD8+ Margin-to-Core Ratio (Spatial Exclusion), median (IQR)	1.4 (0.9–1.8)	4.2 (2.8–6.1)	< 0.001
CD163+ Macrophages (TAMs) (%), mean ± SD	58.5 ± 10.4	76.8 ± 9.2	< 0.001
HIF-1α High Expression, n (%)	82 (34.7%)	160 (67.8%)	< 0.001
CD31+ Microvessel Density (MVD), mean ± SD	35.2 ± 8.5	52.8 ± 11.2	< 0.001
Systemic cytokine profiling (peripheral blood)			
Immunosuppressive cytokines			
Serum TGF-β (pg/mL), median (IQR)	18.2 (12.5–25.4)	32.8 (25.5–45.2)	< 0.001
Serum IL-10 (pg/mL), median (IQR)	12.8 (8.5–18.2)	20.4 (15.2–29.5)	< 0.001
Effector/anti-tumor cytokines			
Serum IFN-γ (pg/mL), median (IQR)	22.5 (14.5–32.5)	11.2 (6.5–18.4)	< 0.001
Systemic cellular Iimmune status			
Peripheral Blood CD4+/CD8+ Ratio, mean ± SD	1.82 ± 0.42	1.25 ± 0.35	< 0.001
Neutrophil-to-Lymphocyte Ratio (NLR) ≥ 4.0, n (%)	68 (28.8%)	147 (62.3%)	< 0.001
Systemic Immune-Inflammation Index (SII) > 600, n (%)	75 (31.8%)	165 (69.9%)	< 0.001

IRJS, Immuno-Radiomic Joint Score; IQR, interquartile range; SD, standard deviation; TAMs, tumor-associated macrophages; HIF-1α, hypoxia-inducible factor 1-alpha.

The CD8^+^ Margin-to-Core Ratio represents the density of CD8^+^ T cells at the invasive margin divided by the density at the tumor core. A higher ratio indicates severe spatial immune exclusion (T cells are trapped outside).

The cohort was stratified into Low and High IRJS Risk groups based on the optimal cutoff value strictly determined from the training cohort. Statistical comparisons were performed using the Student’s *t*-test for normally distributed continuous variables, the Mann-Whitney *U* test for non-normally distributed variables, and the Pearson *χ^2^* test for categorical variables. Bold *P* values indicate statistical significance (*P* < 0.05).

### 3.6 Exploratory survival analysis for immune endpoints and early dynamic remodeling

#### 3.6.1 Predicting primary ICI resistance and long-term benefit

**Figure 6:**
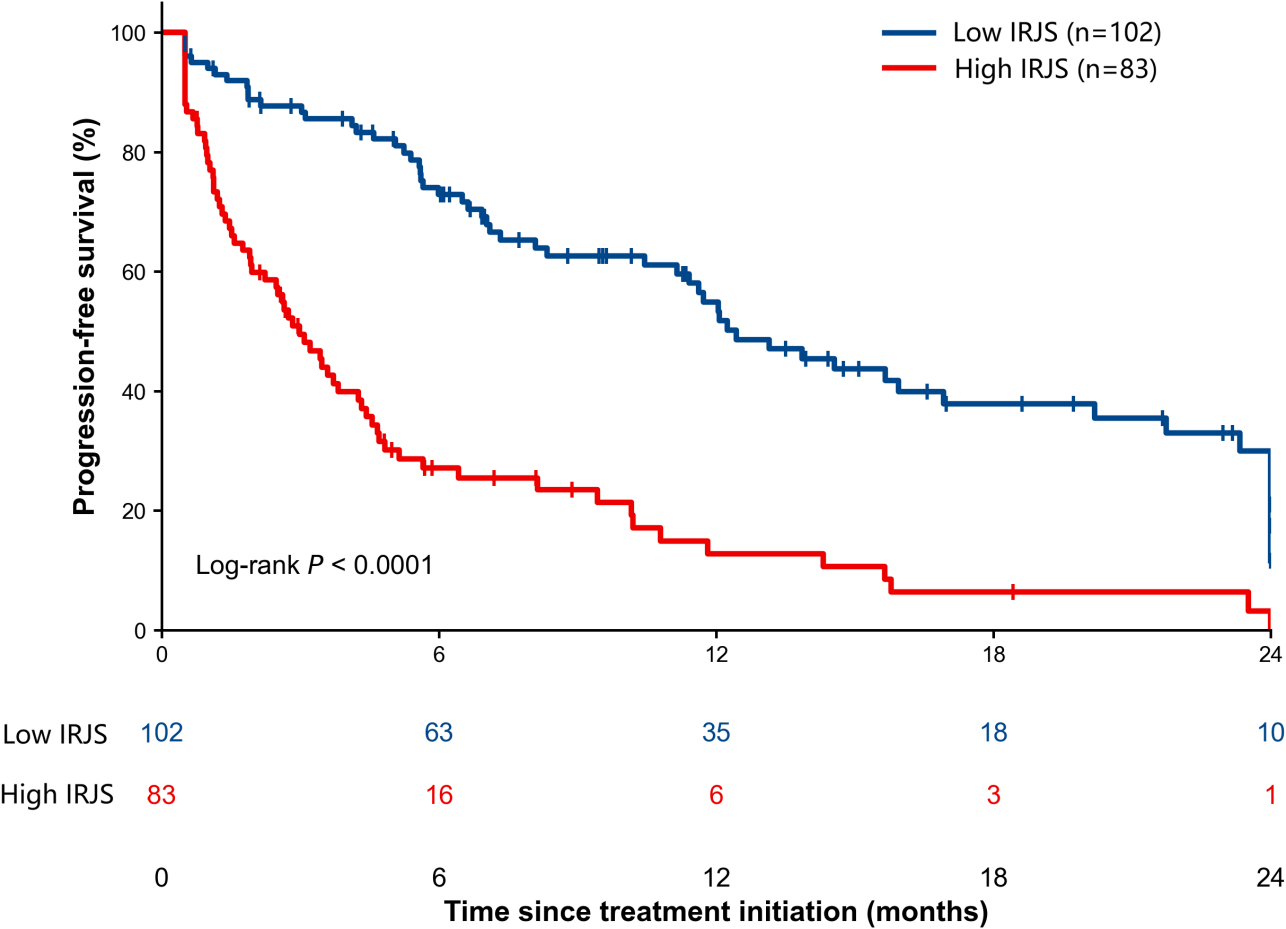
Kaplan-Meier estimates of progression-free survival (PFS) in the immunotherapy validation cohort stratified by the Immuno-Radiomic Joint Score (IRJS). Patients identified as “High IRJS Risk” (red line), indicating severe local physical/metabolic barriers and systemic immune exhaustion, exhibited significantly worse PFS following immune checkpoint inhibitor (ICI) therapy compared to the “Low IRJS Risk” group (blue line). The vertical tick marks indicate censored events. Differences in survival distribution were evaluated using the log-rank test (*P* < 0.001). To avoid threshold-seeking bias, patients were stratified into High IRJS and Low IRJS risk groups purely for visualization purposes based on the median continuous IRJS of this specific cohort (cut-off = 0.15). The log-rank test indicates a significant difference in PFS between the median-stratified groups.

Among the continuous cohort, 185 patients received ICI-based systemic therapy (82 on ICI monotherapy; 103 on ICI combined with anti-angiogenic agents/chemotherapy), the median clinical follow-up time was 24.5 months (95% CI: 21.2–28.1). At the time of data cutoff, 132 progression-free survival (PFS) events (71.4%) had occurred, and 53 patients (28.6%) were censored. Multivariable Cox regression (**Table 5**) confirmed that, after adjusting for covariates such as morphological tumor burden, histological origin, and ICI regimen, an increase in the continuous baseline IRJS score was an independent risk factor predicting primary ICI resistance and shorter progression-free survival (PFS) (HR = 1.23, 95% CI: 1.12–1.27, *P* < 0.001). To avoid *post-hoc* threshold bias when visualizing survival differences within the specific ICI-treated subgroup, we refrained from applying the optimal Youden index cut-off derived from the training set. Instead, for the Kaplan-Meier plot (**Figure 6**), the cohort was stratified using the median continuous IRJS of the ICI group purely for unbiased visualization (cut-off = 0.15). As demonstrated, patients above the median IRJS exhibited significantly shorter PFS.

**Table 5:** Univariate and multivariate Cox proportional hazards regression analysis for progression-Free Survival (PFS) in the immunotherapy subgroup.

Clinical and radiomic variables	Univariate analysis		Multivariate analysis	
	Hazard ratio (95% CI)	P value	Hazard ratio (95% CI)	P value
Demographics & clinical status				
Age (≥ 65 vs.< 65 years)	1.15 (0.82–1.64)	0.412	–	–
Sex (Male vs. Female)	1.08 (0.75–1.52)	0.655	–	–
ECOG Performance Status(2 vs. 0-1)	1.68 (1.05–2.65)	0.031	1.35 (0.85–2.12)	0.198
Morphological tumor burden				
Number of liver lesions(≥ 3 vs.< 3)	1.72 (1.18–2.54)	0.006	1.28 (0.82–1.95)	0.285
Max lesion diameter (≥ 5 cm vs.< 5 cm)	1.65 (1.12–2.45)	0.014	1.15 (0.74–1.78)	0.534
Systemic biomarkers				
Baseline LDH (> Upper Normal Limit vs. Normal)	1.85 (1.25–2.75)	0.002	1.42 (0.95–2.15)	0.085
Baseline NLR (≥ 4.0 vs.< 4.0)	2.10 (1.45–3.10)	< 0.001	1.65 (1.10–2.45)	0.015
Tumor immuno-radiomics				
IRJS Risk Continuous IRJS (per 0.1-unit increase)	1.21 (1.13–1.29)	< 0.001	1.23 (1.12–1.27)	< 0.001

CI, confidence interval; ECOG, Eastern Cooperative Oncology Group; LDH, lactate dehydrogenase; NLR, neutrophil-to-lymphocyte ratio; IRJS, Immuno-Radiomic Joint Score.

Multivariate Cox proportional hazards regression included variables that demonstrated statistical significance (*P* < 0.05) in the univariate analysis. The proportional hazards assumption was strictly verified for all included covariates using Schoenfeld residuals.

To rigorously address potential threshold-seeking bias and preserve maximal statistical power, the traditional binary IRJS risk stratification (High vs. Low) was abandoned in this revised analysis. Instead, the continuous baseline IRJS (evaluated per 0.1-unit increment) was directly incorporated into the Cox proportional hazards regression. This approach confirms the score’s validity as an independent prognostic biological continuum rather than relying on arbitrary dichotomous cut-offs.

#### 3.6.2 Prediction using early dynamic remodeling based on CE-MRI

Dynamic tracking was evaluated in a retrospectively screened subcohort of 80 high IRJS-risk patients with week 3 imaging records. The cohort was stratified based on early physical barrier disruption utilizing landmark analysis (landmark set at week 3) and resetting the PFS starting point to the follow-up scan day to eliminate immortal time bias. Patients who successfully achieved a significant negative drift in dynamic vascular fractal acceleration (Δ*A_fd_*< −0.20) served as the reference group (n = 32; PFS events = 16). Compared to this reference group, patients who failed to achieve this early negative drift (Δ*A_fd_*< −0.20) experienced a significantly higher risk of disease progression (HR = 3.42, 95% CI: 2.18–5.16, P< 0.001), (n = 48; PFS events = 42), as shown in **Supplementary Figure 2**. This confirms that successful physical barrier disruption at week 3 provides a durable and significant protective effect on long-term PFS.

The biological validity of defining the ‘High TIME Barrier’ via median baseline CD8^+^ and PD-L1 expression was empirically confirmed by clinical outcomes. Patients harboring this median-defined phenotype experienced significantly shorter progression-free survival (PFS) upon receiving ICI therapy, validating that this relative statistical threshold effectively maps to genuine primary immunotherapeutic resistance.

#### 3.6.3 Sensitivity analysis

To address potential selection bias and validate the generalizability of this indicator, a parallel assessment utilized routine CE-MRI data from the standard follow-up point (weeks 6–8), (**Supplementary Table 6**). Consistent with the week 3 findings, patients who failed to demonstrate a significant decrease in Δ*A_fd_* at weeks 6–8 maintained a significantly higher risk of progression compared to the negative drift reference group (HR = 2.95, 95% CI: 1.88–4.62, *P<* 0.001). The week-3 cohort exhibited a slightly higher baseline tumor burden and LDH levels, confirming observational selection bias. Therefore, the survival benefits associated with early Δ*A_fd_* physical barrier disruption must be interpreted strictly as an exploratory, hypothesis-generating finding. This dual time-point validation establishes single-modality Δ*A_fd_* dynamic tracking as a robust clinical surrogate marker.

**Figure 7:**
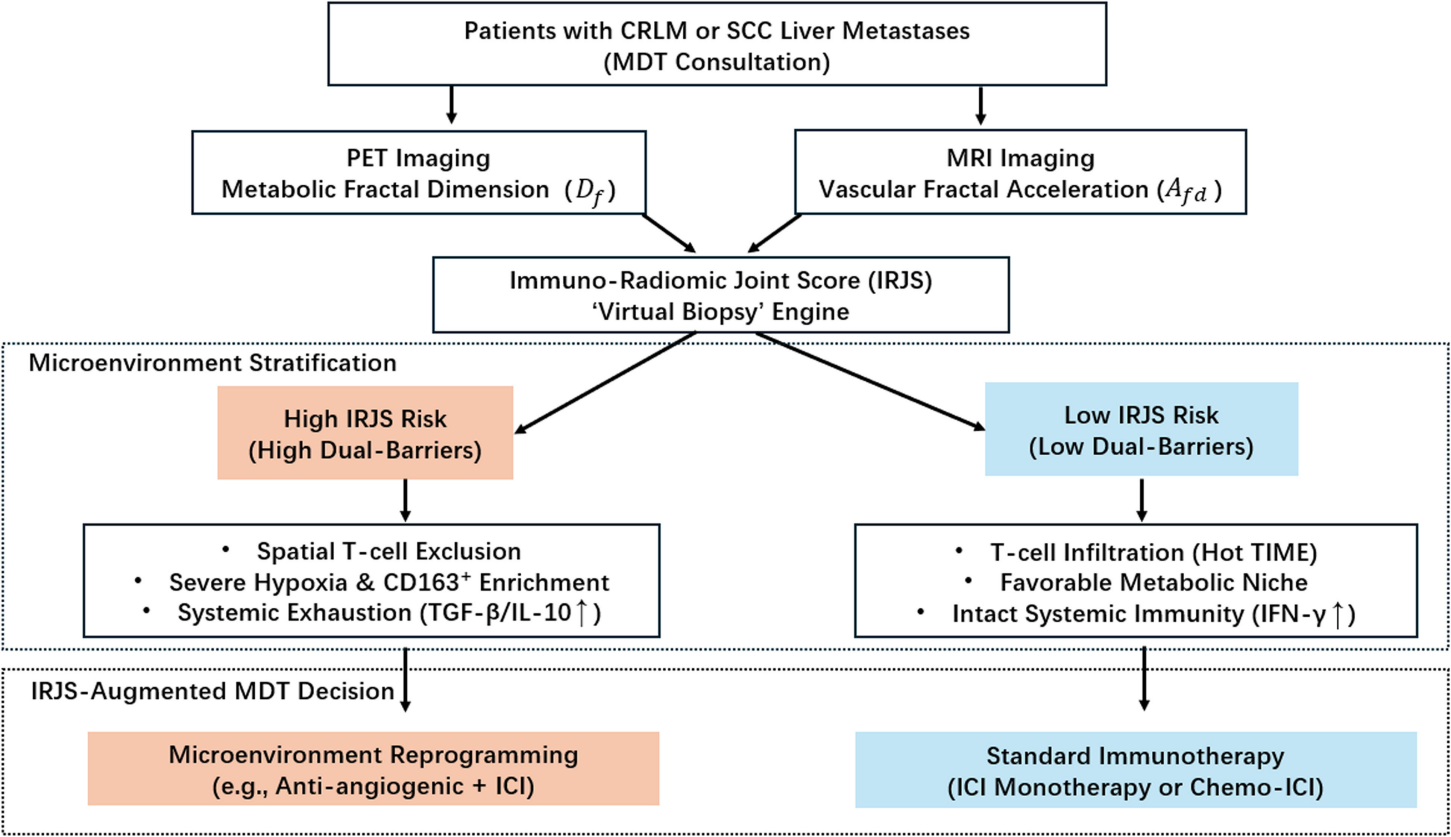
Exploratory clinical translation strategy: A proposed IRJS-guided MDT decision tree. This flowchart illustrates a conceptually proposed, exploratory multidisciplinary team (MDT) workflow utilizing the Immuno-Radiomics Joint Score (IRJS) utilizing the Immuno-Radiomics Joint Score (IRJS) as a non-invasive “virtual biopsy” engine. Upon initial consultation for patients with liver metastases, multi-modal macroscopic fractal dynamics—metabolic fractal dimension (*D_f_*) from ^18^F-FDG PET and vascular fractal acceleration (*A_fd_*) from CE-MRI—are extracted to calculate the IRJS. Based on microenvironment stratification, patients identified as “Low IRJS Risk” exhibit a favorable, “hot” tumor immune microenvironment (TIME) with intact systemic immunity, and are therefore recommended for standard immunotherapy regimens (ICI monotherapy or chemo-immunotherapy). Conversely, patients stratified into the “High IRJS Risk” group possess severe physical-metabolic dual barriers, characterized by significant spatial T-cell exclusion and systemic immune exhaustion (elevated TGF-β/IL-10). To overcome anticipated primary resistance in this highly refractory subgroup, an upfront microenvironment reprogramming strategy (e.g., combining ICIs with anti-angiogenic targeted agents) is recommended to dismantle spatial barriers and restore host anti-tumor immunity. MDT, multidisciplinary team; PET, positron emission tomography; MRI, magnetic resonance imaging; IRJS, Immuno-Radiomics Joint Score; TIME, tumor immune microenvironment; ICI, immune checkpoint inhibitor; TGF-β, transforming growth factor-beta; IL-10, interleukin-10; IFN-γ, interferon-gamma. Note: While the Immuno-Radiomic Joint Score (IRJS) is fundamentally a continuous prognostic biological variable (as demonstrated in **Table 5**), this translational decision tree utilizes a dichotomized approach (High vs. Low IRJS Risk) to facilitate binary clinical actionability. In prospective clinical settings, this stratification threshold could be dynamically calibrated (e.g., using a cohort median or predefined high-risk percentiles) depending on the specific multi-disciplinary intervention goals. Importantly, as this workflow is derived from a retrospective proof-of-concept cohort, it is strictly hypothesis-generating and cannot be applied to direct patient care without rigorous prospective multi-center validation.

To conceptualize future clinical applications, an exploratory, IRJS-guided clinical translation decision tree was constructed (**Figure 7**). This proposed framework is strictly hypothesis-generating and requires prospective validation prior to routine clinical implementation. Standard ICI immunotherapy is recommended for patients identified with low microenvironmental barrier risk. For patients with high IRJS risk, where monotherapy is prone to primary resistance, upfront microenvironmental remodeling strategies are recommended (e.g., combining with anti-angiogenic agents to disrupt the physical-stromal barrier, or using metabolic modulatory interventions) to break spatial barriers and restore systemic anti-tumor immunity.

## 4 Discussion

Our proof-of-concept (PoC) findings indicate that macroscopic fractal dynamics (*A_fd_* and *D_f_*), derived from routine non-invasive imaging, effectively map the physical and metabolic immune barriers of advanced liver metastases. These data establish an *in vivo* quantitative link between spatial heterogeneity and systemic immune exhaustion. Developing the IRJS and the dynamic Δ*A_fd_* monitoring model yields exploratory, hypothesis-generating tools to address primary immunotherapy resistance in solid tumors.

### 4.1 Non-invasive characterization of the physical barrier

Digital pathology in our cohort confirmed that nonlinear increases in MRI vascular fractal acceleration (*A_fd_*) align with the dense proliferation of α-SMA^+^ cancer-associated fibroblasts (CAFs) and abnormal CD31^+^ microvessels. This fibrotic stroma directly restricts CD8^+^ T cells to the tumor margins, enforcing spatial exclusion (19). These macroscopic imaging results support established spatial immunology models; notably, it had been demonstrated that CAF-secreted collagen and TGF-β form the primary physical barrier against T cell infiltration, driving the “immune-excluded” resistance to PD-L1 inhibitors (20). The *A_fd_* metric and its identified nonlinear threshold (*A_fd_* > 1.85) allow us to quantify the closure of this stromal barrier *in vivo*, offering a non-invasive method for early identification of the immune-excluded phenotype.

### 4.2 Systemic immune exhaustion and metabolic reprogramming

While local tumor microenvironments and host systemic immunity were historically viewed in isolation, emerging concepts like the “hepatic immune siphon” (21) suggest liver metastases actively deplete peripheral activated T cells to induce systemic tolerance. Our results expand on this by providing metabolic evidence for this mechanism across spatial scales. Specifically, elevated PET metabolic fractal dimension (*D_f_*) maps directly to local hypoxia (HIF-1α) and glucose competition (GLUT-1), while correlating with CD163^+^ macrophage infiltration and increased peripheral TGF-β. Under conditions of severe hypoxia and metabolic reprogramming, macrophage polarization toward immunosuppressive states is observed. Highly heterogeneous liver metastases strongly linked to elevated systemic TGF-β and depleted anti-tumor factors like IFN-γ. This macroscopic perspective aligns with the hypothesis that advanced liver metastases are strongly coupled with broader systemic immune exhaustion (22).

### 4.3 CRLM and SCC spatial principles and dynamic monitoring

Our data indicate that the spatial heterogeneity of microenvironmental barriers can override the histological origin of the primary tumor. High IRJS scores predicted survival disadvantages in both isolated adenocarcinoma (CRLM) and squamous carcinoma (SCC) cohorts. This observation supports microenvironmental remodeling as a tumor-agnostic therapeutic strategy (23).

Early dynamic fractal drift on routine CE-MRI (Δ*A_fd_*< −0.20 at week 3) reliably predicted prolonged PFS during targeted combination therapy. This reflects the “vascular normalization” theory, where anti-angiogenic agents transiently remodel abnormal vasculature and lower interstitial fluid pressure, temporarily lowering physical barriers to T cell infiltration (24). Tracking dynamic Δ*A_fd_* gives multidisciplinary teams a quantitative, non-invasive method to capture this narrow normalization window in a clinical setting.

Our approach explicitly decouples the radiomic signature from the tumor’s histological origin. Direct cross-cancer training often succumbs to ‘shortcut learning,’ where algorithms merely detect baseline vascular or metabolic differences between adenocarcinomas and squamous carcinomas. By applying subgroup-specific Z-score normalization and anchoring the XGBoost prediction target directly to digital pathology metrics (CD8^+^ and PD-L1), the IRJS was forced to learn a true spatial immune signature. This ensures that a high IRJS reflects a severe microenvironmental barrier relative to the tumor’s own baseline, solidifying its validity as a CRLM and SCC predictor for primary ICI resistance.

### 4.4 Limitations and future perspectives

While this study utilizes cross-scale validation, several constraints must be acknowledged. First, regarding macroscopic folding and single-cell resolution: the fractal dimensions (*A_fd_* and *D_f_*) are voxel-level macroscopic features (1×1×1 mm³). Although they correlate with traditional immunopathological markers, they inherently aggregate complex cellular networks (25). Current digital pathology limits fine resolution of T cell exhaustion (e.g., PD-1/TIM-3/LAG-3 co-expression) or specific suppressive CAF subtypes (26). Future research should co-register macroscopic fractal imaging with spatial transcriptomics and multiplex immunofluorescence (mIF). A spatial multi-omics alignment model could detail the molecular pathways triggered when physical thresholds (e.g., *A_fd_* > 1.85) are crossed. Second, a temporal lag exists in mapping the systemic immune exhaustion. Baseline serum TGF-β and IFN-γ only provide a static view (27), whereas immune remodeling is time-dependent (28). The pharmacokinetic/pharmacodynamic (PK/PD) delay between breaking local barriers and recovering peripheral immunity remains unclear. Prospective longitudinal liquid biopsies are needed to temporally link imaging Δ*A_fd_* changes with circulating tumor DNA (ctDNA) clearance and peripheral blood mononuclear cell (PBMC) single-cell sequencing (29).

Third, our study utilized CD163 as a single immunohistochemical marker to evaluate the immunosuppressive macrophage compartment. While CD163 enrichment robustly correlates with poor prognosis and an immune-tolerant microecology in large clinical cohorts, it is insufficient to definitively map the M1/M2 polarization spectrum (30). Future prospective studies must incorporate multiplex immunofluorescence (mIF) panels (e.g., CD68, CD86, CD163, CD206) and spatial transcriptomics to delineate the precise functional subsets of tumor-associated macrophages influenced by macroscopic metabolic barriers (31, 32).

The correlations between local TIME barriers and peripheral blood cytokines (TGF-β, IL-10, IFN-γ) rely entirely on cross-sectional baseline data. This study establishes an association but cannot prove causality or directionality. It remains equally plausible that pre-existing systemic immune dysregulation in the host facilitates the formation of these local microenvironmental barriers, rather than the tumor actively depleting systemic immunity (33, 34). Definitive proof requires prospective causal modeling and interventional studies, featuring longitudinal pharmacokinetic/pharmacodynamic (PK/PD) profiling and mechanistic *in vivo* evidence, to confirm whether disrupting local spatial barriers with targeted agents directly restores systemic immune fitness (35).

Fourth, while the median-based dichotomization of CD8^+^ density and PD-L1 expression served as a robust, internally controlled threshold to eliminate histological skewing in this proof-of-concept cohort, it remains a cohort-dependent metric. Before this immuno-radiomics framework can be deployed as a universal diagnostic tool, future large-scale, multi-center prospective studies are required to establish absolute, cross-institutional quantitative cutoffs for mapping the High TIME Barrier.

The generalizability of our temporal validation cohort is limited. As both the training (2019–2024) and temporal validation (2025) cohorts were sourced from a single center, they shared highly homogeneous baseline characteristics, demographics, and imaging protocols. A small, highly representative validation set can artificially inflate diagnostic metrics (e.g., AUC) (36, 37). Therefore, the ability of the IRJS to universally predict primary ICI resistance remains unproven across diverse clinical settings. The survival analyses presented in this study must be strictly interpreted as exploratory and hypothesis-generating. Rigorous multi-center external validation is mandatory before these imaging surrogates can be reliably translated into routine clinical practice (38).

The nonlinear thresholds identified by our SHAP analysis (e.g., *A_fd_* > 1.85 and *D_f_* > 2.55) must be interpreted with caution. These values represent mathematical inflection points optimizing the XGBoost model’s predictions within our specific cohort, not proven mechanistic transition states. While they provide valuable exploratory hypotheses regarding when physical and metabolic barriers might critically impair immune infiltration, rigorous independent biological validation—utilizing longitudinal spatial transcriptomics or *in vivo* mechanistic models—is mandatory to confirm whether these algorithm-derived thresholds correspond to true biological bottlenecks.

Finally, the early dynamic cohort evaluating week 3 barrier remodeling (Δ*A_fd_*) relied on retrospective data and is inherently limited by selection bias. Patients receiving ultra-early scans typically presented with specific clinical indications, differing in baseline characteristics from the broader cohort. Prospective longitudinal validation, featuring mandated and uniform imaging intervals, is strictly required to definitively establish week 3 Δ*A_fd_* as an ultra-early warning indicator independent of selection bias.

## 5 Conclusions

This proof-of-concept study positions macroscopic fractal dynamics as a viable, non-invasive method for evaluating the dual microenvironmental barriers in advanced solid tumors. Our analysis links spatial heterogeneity to systemic immune exhaustion across biological scales, demonstrating that dynamic monitoring of physical barrier shifts (Δ*A_fd_*) can highlight early windows for reversing primary therapeutic resistance. While single-cell spatial resolution and cross-platform standardization require further development, this framework translates complex tumor microenvironment theory into quantifiable clinical metrics, offering a translational pathway for tailoring precision immunotherapy across distinct histological subtypes (CRLM and SCC).

## Data Availability

The original contributions presented in the study are included in the article/**Supplementary Material**. Further inquiries can be directed to the corresponding authors.
