# Supplementary material for: Macroscopic fractal dynamics characterize the “physical-metabolic” dual barriers and systemic immune exhaustion associated with primary resistance to immunotherapy in liver metastases
Source: Front Immunol. 2026 Jul 9;17:1878195. doi: 10.3389/fimmu.2026.1878195 (PMC13391586; doi:10.3389/fimmu.2026.1878195)
Supplement: Supplementary file 1 [file Presentation1.pdf]

CLAIM Checklist: Artificial Intelligence in Medical Imaging

Manuscript Title: Macro-Fractal Dynamics Unveil the Dual "Physical-Metabolic" Barriers and Systemic Immune Sink Dictating Primary Immunotherapy Resistance in Liver Metastases

Item	CLAIM Guideline Requirement	Reported in Manuscript (Section / Details)
**Title and Abstract**		
1	**Title:** Indicate that an AI/machine learning model was developed or evaluated.	**Yes.** Addressed in Abstract ("An eXtreme Gradient Boosting (XGBoost) algorithm was utilized to construct an Immuno-Radiomics Joint Score...").
2	**Abstract:** Structured summary including study design, methods, results, and conclusions.	**Yes.** (Background, Methods, Results, Conclusion) with clearly stated AUC and HR metrics.
**Introduction**		
3	**Background:** Scientific and clinical background, including the intended use of the AI model.	**Yes. (Introduction)** Highlights the need for non-invasive TIME assessment and the clinical gap in predicting primary ICI resistance.
4	**Objectives:** Specific objectives and hypotheses of the study.	**Yes. (Introduction)** Three explicit aims provided, including cross-scale mapping, IRJS construction, and dynamic $\Delta A_fd tracking.
**Methods - Data**		
5	**Data sources:** Prospective or retrospective, data sources, and dates of data collection.	**Yes. (Methods 2.1)** Retrospective total cohort (N=472). Training: Mar 2019–Dec 2024. Validation: Jan 2025–Dec 2025.
6	**Eligibility criteria:** Inclusion and exclusion criteria for patients and images.	**Yes. (Methods 2.1 & Figure 1)** Indications for PET/CT, CE-MRI, and baseline IHC availability. Exclusions detailed in Flowchart.
7	**Data preprocessing:** Steps taken to clean, normalize, or harmonize the data.	**Yes. (Methods 2.2 & 2.3)** Histogram matching (PET), N4 bias correction & Z-score (MRI), 1x1x1mm resampling, 3D elastic registration, and **ComBat harmonization** for batch effects.
8	**Data partitions:** How data were divided into training, validation, and testing sets.	**Yes. (Methods 2.1)** Strict chronological split to prevent data leakage (Historical Training vs. Independent Temporal Validation).
**Methods - Ground Truth / Reference**		

Item	CLAIM Guideline Requirement	Reported in Manuscript (Section / Details)
**Standard**		
9	**Reference standard:** How the ground truth was determined (e.g., biopsy, clinical outcome).	**Yes. (Methods 2.4 & 2.10)** IHC (CD8+, CD31, α-SMA, CD163, HIF-1α) for biological phenotypes; iRECIST criteria for PFS endpoints.
10	**Blinding:** Were annotators blinded to clinical outcomes?	**Yes. (Methods 2.2)** Radiologists defining the VOIs were blinded to pathological and clinical outcomes.
**Methods - AI Model**		
11	**Model architecture:** Detailed description of the model or algorithm.	**Yes. (Methods 2.6)** Recursive Feature Elimination (RFE) coupled with eXtreme Gradient Boosting (XGBoost).
12	**Initialization and parameters:** Hyperparameter selection and tuning process.	**Yes. (Methods 2.6 & Supp Table S3)** 10-fold cross-validation combined with grid search. Full parameter space and final values are reported.
13	**Explainability:** Methods used to interpret or explain model predictions.	**Yes. (Methods 2.6 & Results 3.4)** SHAP (SHapley Additive exPlanations) values, global summary plots, and local dependence plots used to discover non-linear thresholds.
**Methods - Evaluation**		
14	**Performance metrics:** Metrics used to evaluate the model (e.g., AUC, sensitivity, specificity).	**Yes. (Methods 2.12)** AUC, ROC, DCA, Kaplan-Meier (Log-rank test), and Hazard Ratios (HR) via multivariate Cox regression.
15	**Statistical analysis:** Methods used to estimate confidence intervals and statistical significance.	**Yes. (Methods 2.12)** 95% CIs reported; FDR correction (Benjamini-Hochberg) for cross-scale correlation matrices; DeLong's test for AUC comparisons.
**Results**		
16	**Data flow:** Flow of patients/images through the study.	**Yes. (Results 3.1 & Figure 1)** Consort/TRIPOD-style flowchart details screening, exclusions, and subset assignments.
17	**Demographics:** Baseline characteristics of the study population in each partition.	**Yes. (Table 1 & Supp Table S1)** Detailed demographics and homogeneity verification between training and validation cohorts.
18	**Model performance:** Results of the AI model on the	**Yes. (Results 3.3 & Table 3)** IRJS achieved training AUC = 0.935 and

Item	CLAIM Guideline Requirement	Reported in Manuscript (Section / Details)
	test/validation sets.	temporal validation AUC = 0.895. Survival predictive value HR = 2.88.
19	**Failure analysis:** Analysis of cases where the model failed or performed poorly.	**Yes. (Results 3.6)** Detailed sub-cohort dynamic sensitivity analysis evaluating edge cases (e.g., pseudo-progression vs. true physical barrier disruption).
**Discussion**		
20	**Key findings:** Summary of main results aligned with the study's objectives.	**Yes. (Discussion)** Confirms non-invasive cross-scale mapping of dual TIME barriers and systemic immune sink.
21	**Clinical implications:** How the AI model could be integrated into clinical workflows.	**Yes. (Discussion & Figure 7)** IRJS-Guided MDT Decision Tree proposed for upfront microenvironment reprogramming.
22	**Limitations:** Acknowledgment of study limitations (bias, generalizability).	**Yes. (Discussion)** Thoroughly addresses macroscopic folding (lack of single-cell granularity), PK/PD temporal lag, single-center bias, and selection bias in dynamic cohorts.
**Other Information**		
23	**Code availability:** Is the code available for peer review or public use?	**Yes. (Methods 2.13)** FRAC-V / FRAC-Navigator plugins and Python scripts are stated to be publicly hosted on GitHub.
24	**Data availability:** Is the dataset accessible?	**Yes. (Methods 2.13)** De-identified datasets will be made available upon request without undue reservation.
25	**Ethics:** IRB approval and informed consent statement.	**Yes. (Materials and Methods 2.1 / Ethics Statement)** Approved by Jinzhou Medical University Ethics Committee (Approval: 2026LL-KY-063).
