# Supplementary material for: Macroscopic fractal dynamics characterize the “physical-metabolic” dual barriers and systemic immune exhaustion associated with primary resistance to immunotherapy in liver metastases
Source: Front Immunol. 2026 Jul 9;17:1878195. doi: 10.3389/fimmu.2026.1878195 (PMC13391586; doi:10.3389/fimmu.2026.1878195)
Supplement: Supplementary file 3 [file Presentation3.pdf]

TRIPOD Statement: Checklist for Prediction Model Development and Validation

Item	Section/ Topic	Checklist Item Description	Reported in Manuscript (Section / Details)	Page
**Title and Abstract**				
1	**Title**	Identify the study as developing and/or validating a multivariable prediction model, the target population, and the outcome to be predicted.	**Yes.** Title identifies the target population (liver metastases) and outcome (primary immunotherapy resistance).	**1**
2	**Abstract**	Provide a summary of objectives, study design, setting, participants, sample size, predictors, outcome, statistical analysis, results, and conclusions.	**Yes. (Abstract)** Clearly structured into Background, Methods, Results, and Conclusion with sample size (N=472) and key metrics (AUC, HR).	**1**
**Introduction**				
3a	**Background**	Explain the medical context (including whether diagnostic or prognostic) and rationale for developing or validating the multivariable prediction model.	**Yes. (Introduction)** Explains the clinical gap in non-invasive TIME assessment and the need to predict primary ICI resistance.	**3**
3b	**Objectives**	Specify the objectives, including whether the study describes the development or	**Yes. (Introduction)** Explicitly states the aims to construct (development) and evaluate (temporal	4

Item	Section/ Topic	Checklist Item Description	Reported in Manuscript (Section / Details)	Page
		validation of the model or both.	validation) the IRJS.	
**Methods**				
4a	**Source data**	Describe the study design or source of data (e.g., randomized trial, cohort, or registry data), separately for the development and validation data sets, if applicable.	**Yes. (Methods 2.1)** Retrospective, observational cohort study using a standardized institutional tumor biobank.	**5**
4b	**Source data**	Specify the key study dates, including start of accrual; end of accrual; and, if applicable, end of follow-up.	**Yes. (Methods 2.1)** Training cohort: Mar 2019–Dec 2024. Independent validation cohort: Jan–Dec 2025.	**5**
5a	**Participants**	Specify key elements of the study setting (e.g., primary care, secondary care, general population) including number and location of centres.	**Yes. (Methods 2.1)** Single-center (First Affiliated Hospital of Jinzhou Medical University).	**5**
5b	**Participants**	Describe eligibility criteria for participants.	**Yes. (Methods 2.1)** Consecutive patients with CRLM or SCC liver metastases, required systemic 18F-FDG PET/CT, CE-MRI, and baseline IHC.	**5**
5c	**Participants**	Give details of treatments received, if relevant.	**Yes. (Methods 2.8 & 2.10)** Subgroup analyses specified for patients receiving ICI monotherapy or	**11**

Item	Section/ Topic	Checklist Item Description	Reported in Manuscript (Section / Details)	Page
			combination regimens.	
6a	**Outcome**	Clearly define the outcome that is predicted by the prediction model, including how and when assessed.	**Yes. (Methods 2.10)** Primary endpoint is Progression-Free Survival (PFS), confirmed via iRECIST criteria.	**12**
6b	**Outcome**	Report any actions to blind assessment of the outcome to be predicted.	**Yes. (Methods 2.2)** Radiologists delineating VOIs were strictly blinded to pathological and clinical outcomes.	**6**
7a	**Predictors**	Clearly define all predictors used in developing or validating the multivariable prediction model, including how and when measured.	**Yes. (Methods 2.3)** Detailed extraction protocols for 3D vascular fractal acceleration (A_{fd}) and metabolic fractal dimension (D_f).	**7**
7b	**Predictors**	Report any actions to blind assessment of predictors for the outcome and other predictors.	**Yes. (Methods 2.4 & 2.5)** IHC and PPP cytokine profiling (ELISA) were performed double-blindly.	**8-9**
8	**Sample size**	Explain how the study size was arrived at.	**Yes. (Methods 2.1)** Consecutive enrollment of all eligible patients during the specified timeframes (N=472).	**5**
9	**Missing data**	Describe how missing data were handled (e.g., complete-case	**Yes. (Methods 2.2 & 2.8)** Complete-case analysis; patients with severe image	**7**

Item	Section/ Topic	Checklist Item Description	Reported in Manuscript (Section / Details)	Page
		analysis, single imputation, multiple imputation) with details of any imputation method.	artifacts or missing ultra-early MRI sequences were strictly excluded.	
10a	**Analysis**	Describe how predictors were handled in the analyses.	**Yes. (Methods 2.6)** Recursive Feature Elimination (RFE) used to screen core fractal parameters.	**10**
10b	**Analysis**	Specify type of model, all model-building procedures (including any predictor selection), and method for internal validation.	**Yes. (Methods 2.6)** XGBoost algorithm, 10-fold cross-validation, and exhaustive grid search.	**10**
10c	**Analysis**	For validation, describe how the predictions were calculated.	**Yes. (Methods 2.7)** XGBoost continuous predicted probability (0-1) defined as IRJS; locked threshold applied to validation set.	**11**
10d	**Analysis**	Specify all measures used to assess model performance and, if relevant, to compare multiple models.	**Yes. (Methods 2.12)** Concordance evaluated via AUC (ROC), DCA for clinical utility, and HR via multivariate Cox regression.	**13**
10e	**Analysis**	Describe any model updating (e.g., recalibration) arising from the validation, if done.	**Not Applicable.** The model weights and thresholds were strictly locked to avoid overfitting (Methods 2.7); no recalibration was performed.	**NA**

Item	Section/ Topic	Checklist Item Description	Reported in Manuscript (Section / Details)	Page
**Results**				
13a	**Participants**	Describe the flow of participants through the study, including the number of participants with and without the outcome and, if applicable, a summary of the follow-up time.	**Yes. (Results 3.1 & Figure 1)** Consort-style flowchart outlines screening, exclusions, and assignment to training/validation and targeted sub-cohorts.	**14**
13b	**Participants**	Describe the characteristics of the participants (basic demographics, clinical features, available predictors), including the number of participants with missing data for predictors and outcome.	**Yes. (Table 1 & Supp Table S1)** Comprehensive breakdown of baseline characteristics across cohorts.	**14**
13c	**Participants**	For validation, show a comparison with the development data of the distribution of important variables (demographics, predictors and outcome).	**Yes. (Results 3.1 & Supp Table S1)** Confirmed no statistical differences between training and validation cohorts regarding baseline traits.	**14**
14a	**Model dev.**	Specify the number of participants and outcome events in each analysis.	**Yes. (Results 3.6 & Table 5)** PFS analysis specifically conducted on the 185 patients receiving systemic ICI therapy.	**18,41**
14b	**Model**	If done, report the	**Yes. (Table 5)** Univariate	**41**

Item	Section/ Topic	Checklist Item Description	Reported in Manuscript (Section / Details)	Page
	**dev.**	unadjusted association between each candidate predictor and outcome.	Cox regression results provided alongside multivariate results.	
15a	**Specification**	Present the full prediction model to allow predictions for individuals (i.e., all regression coefficients, and model intercept or baseline survival at a given time point).	**Yes. (Methods 2.6 & Results 3.4)** Full parameter space detailed in Supp Table S3; global and local feature weights resolved via SHAP framework.	**10,17**
15b	**Specification**	Explain how to use the prediction model.	**Yes. (Methods 2.6 & Figure 7)** IRJS calculation mechanism explained, ending with a translational MDT decision tree.	**10,47**
16	**Performance**	Report performance measures (with CI) for the prediction model.	**Yes. (Results 3.3)** Training AUC = 0.935; Temporal Validation AUC = 0.895.	**16**
17	**Validation**	If validating, report performance measures (with CI) for the prediction model in the validation data.	**Yes. (Results 3.3, Figure 4)** Validated predictive power and clinical net benefit (DCA) reported for the 2025 cohort.	**16,45**
**Discussion**				
18	**Limitations**	Discuss any limitations of the study (such as non-representative sample, few events per predictor, missing data).	**Yes. (Discussion)** Thoroughly addresses macroscopic folding (lack of single-cell granularity), PK/PD temporal lag, single-center bias, and selection bias.	**20**

Item	Section/ Topic	Checklist Item Description	Reported in Manuscript (Section / Details)	Page
19a	**Interpretation**	For validation, discuss the results with reference to performance in the development data, and any other validation data.	**Yes. (Discussion)** Generalizability verified and confirmed across independent temporal dataset and specific pure histology sub-cohorts.	**20**
19b	**Interpretation**	Give an overall interpretation of the results, considering objectives, limitations, results from similar studies, and other relevant evidence.	**Yes. (Discussion)** Validates findings against classical theories (e.g., Mariathasan et al., Jain RK et al.) regarding the immune sink and vascular normalization.	**20**
20	**Implications**	Discuss the potential clinical use of the model and implications for future research.	**Yes. (Discussion & Figure 7)** Suggests upfront microenvironment reprogramming (anti-angiogenics) guided by IRJS.	**22,47**
**Other**				
21	**Supp. info**	Provide information about the availability of supplementary resources, such as study protocol, Web calculator, and data sets.	**Yes. (Methods 2.13)** De-identified data availability stated; FRAC plugins and scripts publicly hosted on GitHub.	**24**
22	**Funding**	Give the source of funding and the role of the funders for the present study.	**Yes. (Title Page)** Funding statement included.	**23**
