# Supplementary material for: Macroscopic fractal dynamics characterize the “physical-metabolic” dual barriers and systemic immune exhaustion associated with primary resistance to immunotherapy in liver metastases
Source: Front Immunol. 2026 Jul 9;17:1878195. doi: 10.3389/fimmu.2026.1878195 (PMC13391586; doi:10.3389/fimmu.2026.1878195)
Supplement: Supplementary file 4 [file Table1.docx]

**Supplementary Appendix**

**Supplementary Methods: Computational Reproducibility Specifications**

To ensure full transparency and independent reproducibility of the computational workflows utilized in this study, the exact software environments, hyperparameter configurations, and algorithmic pseudocodes for the custom FRAC-V module and the machine learning pipeline are detailed below.

**1. Software Environment and Dependencies**

All image processing, fractal extraction, and machine learning model training were executed in a standardized Python environment.

**Operating System:** Ubuntu 20.04 LTS (64-bit) / Windows 10 Pro

**Programming Language:** Python version 3.9.7

**Core Libraries & Versions:**

SimpleITK (v2.1.1): Used for 3D NIfTI/DICOM read/write, voxel resampling, and spatial transformations.

scikit-image (v0.18.3): Used for morphological operations and bounding box extractions.

numpy (v1.21.2) & scipy (v1.7.1): Used for matrix operations, logarithmic regressions, and statistical tests.

scikit-learn (v1.0.2): Used for data normalization, logistic regression baseline, and cross-validation splitting.

xgboost (v1.5.0): Used for the construction of the Immuno-Radiomics Joint Score (IRJS) model.

shap (v0.40.0): Used for local/global explainability and threshold dependency analysis.

**2. Pseudocode for the FRAC-V Extraction Module**

The FRAC-V module computes multidimensional macroscopic fractal dynamics. Algorithm 1 describes the 3D finite-resolution box-counting method used to extract the PET metabolic fractal dimension ($D_{f}$). Algorithm 2 describes the extraction of dynamic MRI vascular fractal metrics ($\Delta A_{fd}$ and $A_{fd}$).

**Algorithm 1: 3D Box-Counting for PET Metabolic Fractal Dimension** ($D_{f}$)

**Input:** * PET_Image: 3D registered PET volume (resampled to 1×1×1 mm³).

- Tumor_Mask: 3D binary mask of the target lesion.
- SUV_bg: Mean SUV of the normal liver background.

**Output:** * Df: 3D Metabolic Fractal Dimension.

- R_squared: Goodness-of-fit for scale-invariance validation.

1: // Step 1: Binarization based on metabolic threshold

2: Threshold = SUV_bg + 2 * Standard_Deviation(Background)

3: Binary_Metabolic_Core = (PET_Image >= Threshold) AND (Tumor_Mask == 1)

4:

5: // Step 2: Define logarithmic box sizes for 3D grid

6: Box_Sizes (r) = [2, 4, 8, 16, 32] // in mm (voxels)

7: Box_Counts (N) = Empty Array

8:

9: // Step 3: Box-Counting Execution

10: FOR EACH r IN Box_Sizes DO

11: Grid 3D space into non-overlapping boxes of size (r x r x r)

12: Count = 0

13: FOR EACH box IN Grid DO

14: IF box contains at least one voxel where Binary_Metabolic_Core == 1 THEN

15: Count = Count + 1

16: END IF

17: END FOR

18: Append Count to Box_Counts (N)

19: END FOR

20:

21: // Step 4: Fractal Dimension Calculation via Log-Log Regression

22: X = log(1 / Box_Sizes)

23: Y = log(Box_Counts)

24: Df, Intercept, R_squared = Linear_Regression(X, Y)

25:

26: // Step 5: Quality Control

27: IF R_squared < 0.95 THEN

28: Flag lesion as "Scale-Variant / Invalid"

29: END IF

30: RETURN Df, R_squared

**Algorithm 2: Dynamic MRI Vascular Fractal Acceleration ($A_{fd}$)**

**Input:**

- MRI_Baseline, MRI_Arterial, MRI_Portal: 3D registered CE-MRI phases (Unenhanced, 30s, and 70s).
- Tumor_Mask: 3D binary mask of the target lesion.

**Output:**

- V_fd: Vascular Fractal Velocity (wash-in phase).
- A_fd: Vascular Fractal Acceleration.

1: // Step 1: Extract Static Vascular Fractal Dimensions per phase

2: Df_Baseline = Calculate_3D_Box_Counting(MRI_Baseline, Tumor_Mask, Enhancement_Threshold)

3: Df_Arterial = Calculate_3D_Box_Counting(MRI_Arterial, Tumor_Mask, Enhancement_Threshold)

4: Df_Portal = Calculate_3D_Box_Counting(MRI_Portal, Tumor_Mask, Enhancement_Threshold)

5:

6: // Step 2: Calculate Spatiotemporal Gradients

7: // Assuming normalized temporal intervals (e.g., Δt adjusted based on scanning protocol)

8: V_fd_early = Df_Arterial - Df_Baseline // Contrast wash-in velocity

9: V_fd_late = Df_Portal - Df_Arterial // Transitional perfusion velocity

10:

11: // Step 3: Compute Vascular Acceleration

12: A_fd = V_fd_late - V_fd_early

13: RETURN V_fd_early (as V_fd), A_fd

**3. XGBoost Machine Learning Pipeline and Hyperparameter Grid**

To prevent data leakage, hyperparameter tuning was strictly confined to the training cohort (2019–2024, n=400). We employed an exhaustive grid search optimized via 10-fold cross-validation. The optimization objective function was set to binary:logistic and evaluated based on maximizing the Area Under the Curve (AUC).

**1. Hyperparameter Search Space:**

The GridSearchCV explicitly explored the following parameter boundaries:

- max_depth: [3, 4, 5, 6] *(Controls tree complexity and prevents overfitting)*
- learning_rate (eta): [0.01, 0.05, 0.1, 0.2] *(Step size shrinkage)*
- n_estimators: [100, 200, 300, 500] *(Number of boosting rounds)*
- subsample: [0.7, 0.8, 1.0] *(Fraction of observations to be randomly sampled for each tree)*
- colsample_bytree: [0.7, 0.8, 1.0] *(Fraction of features used per tree)*
- gamma (min_split_loss): [0, 0.1, 0.5] *(Minimum loss reduction required to make a further partition)*
- min_child_weight: [1, 3, 5] *(Minimum sum of instance weight needed in a child)*

**2. Final Optimal Configuration (IRJS Model):**

The configuration that yielded the highest cross-validated AUC without exhibiting substantial variance across folds was locked for the final model generation and subsequent independent validation:

- max_depth: 4
- learning_rate: 0.05
- n_estimators: 200
- subsample: 0.8
- colsample_bytree: 0.8
- gamma: 0.1
- min_child_weight: 3
- objective: 'binary:logistic'
- eval_metric: 'auc'

No post-training calibration or threshold tweaking was performed on the independent 2025 temporal validation cohort, ensuring the reported generalizability is fully authentic.

**Supplementary Flowchart**

**Patients with suspected liver metastases screened (N = 545)**

**Excluded (n = 73)**

**• Missing baseline ^18^F-FDG PET/CT or CE-MRI (n = 31)**

**• No baseline core biopsy or surgical IHC available (n = 24)**

**• Severe image artifacts or poor image quality (n = 12)**

**• Incomplete clinical follow-up data (n = 6)**

**Targeted Analysis Sub-cohorts (from Total Cohort)**

**Immunotherapy (ICI) Subgroup**

**(Receiving ICI-based therapy)**

**n = 185**

**Used for Efficacy & Survival Analysis**

**Dynamic Tracking Subgroup**

**(High-risk with Week 3 MRI)**

**n = 80**

**Used for Barrier Reprogramming**

**AnalysisUsed for Efficacy & Survival Analysis**

**Final Enrolled Cohort (N = 472):**

**• CRLM (Adenocarcinoma): n = 195**

**• SCC (Squamous): n = 277**

**ICI Combination Therapy**

**[ICI + Anti-angiogenic/Chemo]**

**n = 103**

**Treatment Modality Stratification (from Total Cohort)**

**n=472**

**Standard Targeted/Chemotherapy Only**

**n = 287**

**ICI Monotherapy**

**n = 82**

**Historical Training Cohort**

**(Mar 2019 – Dec 2024)**

**n = 400**

**Independent Validation Cohort**

**(Jan 2025 – Dec 2025)**

**n = 72**

**Flowchart Notes:**

**Total Enrolled (N=472):** Represents the main cohort used for cross-scale mapping and IRJS construction.

**CRLM and SCC:** Serve as biological models for metabolic and physical barriers, respectively.

**Temporal Validation:** 2025 data were strictly locked and used only for model evaluation to ensure zero data leakage.

**ICI Subgroup:** Includes patients receiving either ICI monotherapy (n=82) or combo therapy (n=103).

**Dynamic Tracking Subgroup:** Consists of high IRJS-risk patients with available ultra-early imaging at week 3.

**Supplementary Flowchart.** Patient enrollment, exclusion cascade, and sub-cohort assignment. The flowchart explicitly details the consecutive screening of 545 patients, enumerating the specific reasons for the exclusion of 73 patients to verify the absence of intentional selection bias. The final enrolled cohort (N=472) is stratified by primary histological origin (CRLM and SCC) and subsequently by systemic treatment modalities. The ICI-treated subgroup (n=185), comprising both monotherapy and combination therapy, was utilized for the primary progression-free survival (PFS) analyses.

**Supplementary Tables**

**Supplementary Table S1. Baseline characteristics comparing the historical training cohort and the independent temporal validation cohort.**

| **Characteristics** | **Training Cohort(Mar 2019 – Dec 2024, n = 400)** | **Validation Cohort(Jan 2025 – Dec 2025, n = 72)** | **P Value** |
| --- | --- | --- | --- |
| **Demographics** |  |  |  |
| Age (years), mean ± SD | 63.6 ± 9.7 | 63.1 ± 10.2 | 0.684 |
| Sex (Male), n (%) | 264 (66.0%) | 48 (66.7%) | 0.912 |
| **Baseline Tumor Burden** |  |  |  |
| Number of liver metastases, median (IQR) | 3 (2–5) | 3 (2–4) | 0.588 |
| Max diameter of lesions (cm), mean ± SD | 4.5 ± 1.8 | 4.4 ± 1.7 | 0.652 |
| **Primary Tumor Histology** |  |  | 0.845 |
| - CRLM, n (%) | 166 (41.5%) | 29 (40.3%) |  |
| - SCC, n (%) | 234 (58.5%) | 43 (59.7%) |  |
| **Systemic Treatment Regimen** |  |  | 0.956 |
| - ICI Monotherapy, n (%) | 69 (17.3%) | 13 (18.1%) |  |
| - ICI Combo, n (%) | 88 (22.0%) | 15 (20.8%) |  |
| - Targeted/Chemotherapy only, n (%) | 243 (60.8%) | 44 (61.1%) |  |

**Table Notes:** Continuous variables are presented as mean ± standard deviation (SD) or median (interquartile range, IQR), and compared using the Student's *t*-test or Mann-Whitney *U* test, as appropriate. Categorical variables are expressed as counts (percentages) and compared using the Chi-square (*χ*^2^) test. All *P* values > 0.05 indicate no significant differences between the historical training cohort and the independent validation cohort, confirming the absence of temporal drift or selection bias.

**Abbreviations:** CRLM, colorectal liver metastases; SCC, squamous cell carcinoma; ICI, immune checkpoint inhibitor.

**Supplementary Table S2. Antibody specifications and standardized immunohistochemistry (IHC) protocols.**

| Target Protein | Clone | Vendor | Host | Dilution | Antigen Retrieval | Incubation |
| --- | --- | --- | --- | --- | --- | --- |
| CD8 | SP57 | Roche Ventana, USA | Rabbit | RTU | Tris-EDTA (pH 8.5), 95°C, 36 min | 37°C, 32 min |
| PD-L1 | 22C3 | Agilent Dako, USA | Mouse | RTU | Tris-EDTA (pH 9.0), 97°C, 20 min | 25°C, 60 min |
| CD31 | JC70A | Agilent Dako, USA | Mouse | 1:50 | Tris-EDTA (pH 9.0), 95°C, 20 min | 37°C, 30 min |
| ⍺-SMA | 1A4 | Abcam, UK | Mouse | 1:200 | Citrate Buffer (pH 6.0), 95°C, 20 min | 37°C, 30 min |
| CD163 | EPR19518 | Abcam, UK | Rabbit | 1:500 | Tris-EDTA (pH 9.0), 95°C, 20 min | 37°C, 30 min |
| GLUT-1 | EPR3915 | Abcam, UK | Rabbit | 1:250 | Citrate Buffer (pH 6.0), 95°C, 20 min | 37°C, 30 min |
| HIF-1⍺ | EP1215Y | Abcam, UK | Rabbit | 1:100 | Tris-EDTA (pH 9.0), 95°C, 30 min | 37°C, 60 min |

**Abbreviations:** RTU, Ready-to-use; ⍺-SMA, Alpha-smooth muscle actin; HIF-1⍺, Hypoxia-inducible factor 1-⍺.

**Methodological Standardization:**

**Automated Staining Systems:** CD8 staining was performed on the BenchMark ULTRA automated platform (Roche Diagnostics) utilizing the ultraView Universal DAB Detection Kit. Staining for PD-L1 (22C3 pharmDx) and other biomarkers was executed on the Autostainer Link 48 platform (Agilent Dako) via the EnVision FLEX visualization system to minimize manual batch-effects.

**Antigen Retrieval and Controls:** Tris-EDTA refers to Target Retrieval Solution (High pH). Citrate Buffer refers to Target Retrieval Solution (Low pH 6.0). Appropriate positive controls (e.g., tonsil for CD8/CD163, placenta for GLUT-1) and negative controls were included in each run to ensure staining specificity.

**Supplementary Table S3. Final optimized hyperparameters and grid search space for the XGBoost model.**

| Hyperparameter | Description | Search Space (Grid) | Final Optimized Value |
| --- | --- | --- | --- |
| n_estimators | Number of boosting rounds (trees). | [100, 200, 300, 400, 500] | 300 |
| learning_rate | Step size shrinkage to prevent overfitting. | [0.01, 0.05, 0.1, 0.2] | 0.05 |
| max_depth | Maximum depth of a tree; controls complexity. | [3, 4, 5, 6] | 4 |
| min_child_weight | Minimum sum of instance weight in a child. | [1, 3, 5, 7] | 3 |
| subsample | Ratio of training instances per tree. | [0.6, 0.7, 0.8, 0.9] | 0.8 |
| colsample_bytree | Subsample ratio of columns per tree. | [0.6, 0.7, 0.8, 0.9] | 0.8 |
| gamma | Minimum loss reduction for further partitioning. | [0, 0.1, 0.2, 0.5] | 0.1 |
| reg_alpha | L1 regularization term on weights. | [0, 0.1, 0.5, 1.0] | 0.5 |
| reg_lambda | L2 regularization term on weights. | [0.1, 1.0, 2.0, 5.0] | 1.0 |
| objective | Learning task and objective function. | Fixed | binary:logistic |

**Table Notes:** Optimization Strategy: Optimal hyperparameters were determined through 10-fold cross-validation combined with an exhaustive grid search on the training cohort (2019-2024). The objective function was set to binary: logistic to output continuous predicted probabilities of microenvironmental barriers (defined as IRJS). To mitigate the risk of radiomic overfitting, tree complexity (max_depth) was restrained to 4, and significant L1/L2 regularization penalties were applied.

**Supplementary Table S4. Performance Comparison of Multiple Machine Learning Algorithms for Predicting the High TIME Barrier Phenotype in the Training Cohort (2019-2024, n = 400)**

| **Algorithm** | **AUC (95% CI)** | **Accuracy (%)** | **Sensitivity (%)** | **Specificity (%)** | **F1-Score** |
| --- | --- | --- | --- | --- | --- |
| Logistic Regression (LR) | 0.792 (0.745–0.839) | 75.2 | 72.4 | 78.0 | 0.738 |
| Support Vector Machine (SVM) | 0.815 (0.772–0.858) | 78.5 | 76.8 | 80.2 | 0.776 |
| Random Forest (RF) | 0.895 (0.860–0.930) | 84.8 | 82.5 | 87.1 | 0.841 |
| Multi-Layer Perceptron (MLP)* | 0.875 (0.835–0.915) | 86.0 | 85.2 | 86.8 | 0.858 |
| LightGBM | 0.928 (0.902–0.954) | 86.8 | 85.5 | 88.1 | 0.865 |
| **XGBoost (IRJS Model)** | **0.935 (0.912–0.958)** | **87.5** | **88.2** | **86.5** | **0.878** |

**Notes:**

All models were evaluated using 10-fold cross-validation within the training cohort.

The features inputted into all algorithms were identical (subgroup-normalized $D_{f}$ and $A_{fd}$, alongside selected clinical baseline features).

*The Multi-Layer Perceptron (MLP) deep learning model consisted of 3 hidden layers (128, 64, 32 neurons) with ReLU activation and Dropout regularization. Despite high overall accuracy, its lower AUC relative to tree-based ensembles suggests a propensity for overfitting on this structured tabular dataset.

**AUC:** Area Under the Receiver Operating Characteristic Curve; **CI:** Confidence Interval.

Bold text indicates the optimal performing model selected for the final Immuno-Radiomics Joint Score (IRJS).

**Supplementary Table S5. Confusion Matrices of the Immuno-Radiomics Joint Score (IRJS) in the Training and Temporal Validation Cohorts.**

**(A) Training Cohort (2019–2024, n = 400)**

|  | **Predicted: High TIME Barrier** | **Predicted: Low TIME Barrier** | **Total** |
| --- | --- | --- | --- |
| **Actual: High TIME Barrier** | **202** (True Positive) | **27** (False Negative) | 229 |
| **Actual: Low TIME Barrier** | **23** (False Positive) | **148** (True Negative) | 171 |
| **Total** | 225 | 175 | 400 |

Derived Metrics (Training):

- Sensitivity = 202 / 229 = 88.2%
- Specificity = 148 / 171 = 86.5%
- Overall Accuracy = 350 / 400 = 87.5%

**(B) Independent Temporal Validation Cohort (2025, n = 72)**

|  | **Predicted: High TIME Barrier** | **Predicted: Low TIME Barrier** | **Total** |
| --- | --- | --- | --- |
| **Actual: High TIME Barrier** | **38** (True Positive) | **6** (False Negative) | 44 |
| **Actual: Low TIME Barrier** | **4** (False Positive) | **24** (True Negative) | 28 |
| **Total** | 42 | 30 | 72 |

Derived Metrics (Validation):

- Sensitivity = 38 / 44 = 86.4%
- Specificity = 24 / 28 = 85.7%
- Overall Accuracy = 62 / 72 = 86.1%

(Note: The cutoff threshold applied to the validation cohort was strictly locked from the training cohort's maximum Youden index, ensuring zero data leakage).

**Supplementary Table S6. Baseline Characteristics Comparison Between the Week-3 Dynamic Imaging Subcohort and the Remaining ICI-Treated Cohort**

| **Characteristics** | **Week-3 Dynamic Imaging Subcohort (n=80)** | **Remaining ICI-Treated Cohort (n=105)** | ***P* Value** |
| --- | --- | --- | --- |
| **Demographics** |  |  |  |
| Age (years), mean ± SD | 62.5 ± 9.4 | 63.1 ± 10.1 | 0.672 |
| Sex (Male/Female), n | 55 / 25 | 70 / 35 | 0.765 |
| ECOG Performance Status (0-1 / 2), n | 62 / 18 | 86 / 19 | 0.428 |
| Histological Phenotype (CRLM / SCC), n | 35 / 45 | 47 / 58 | 0.865 |
| **Morphological Tumor Burden** |  |  |  |
| Number of liver lesions, median (IQR) | 4 (2–6) | 3 (1–5) | **0.045** |
| Max lesion diameter (cm), mean ± SD | 5.1 ± 1.8 | 4.4 ± 1.6 | **0.006** |
| **Baseline Serum Markers** |  |  |  |
| Baseline Serum LDH (U/L), median (IQR) | 285 (210–340) | 240 (180–310) | **0.021** |
| Neutrophil-to-Lymphocyte Ratio (NLR) > 4.0, n (%) | 45 (56.2%) | 48 (45.7%) | 0.154 |
| Systemic Immune-Inflammation Index (SII) > 600, n (%) | 48 (60.0%) | 51 (48.6%) | 0.118 |
| **Macroscopic Fractal Probes** |  |  |  |
| Baseline IRJS Risk (High Dual-Barrier), n (%) | 60 (75.0%) | 72 (68.6%) | 0.335 |

**Abbreviations:** **SD**, standard deviation; **IQR**, interquartile range; **ECOG**, Eastern Cooperative Oncology Group; **CRLM**, colorectal liver metastases; **SCC**, squamous cell carcinoma; **LDH**, lactate dehydrogenase; **IRJS**, Immuno-Radiomic Joint Score.

**Note:** The Week-3 Dynamic Imaging Subcohort consists of patients who underwent an ultra-early contrast-enhanced MRI at 3 weeks post-treatment initiation. The Remaining ICI-Treated Cohort consists of patients who followed standard clinical imaging intervals (6–8 weeks). Statistical comparisons were performed using the Student’s *t*-test for normally distributed continuous variables, the Mann-Whitney *U* test for non-normally distributed continuous variables, and the Pearson's chi-square test for categorical variables.

Bold P values indicate statistical significance (*P* < 0.05), quantitatively confirming the presence of observational selection bias (higher baseline morphological tumor burden and elevated LDH) in the ultra-early imaging cohort, which aligns with clinical indications for premature efficacy evaluation.

**Supplementary Figures**


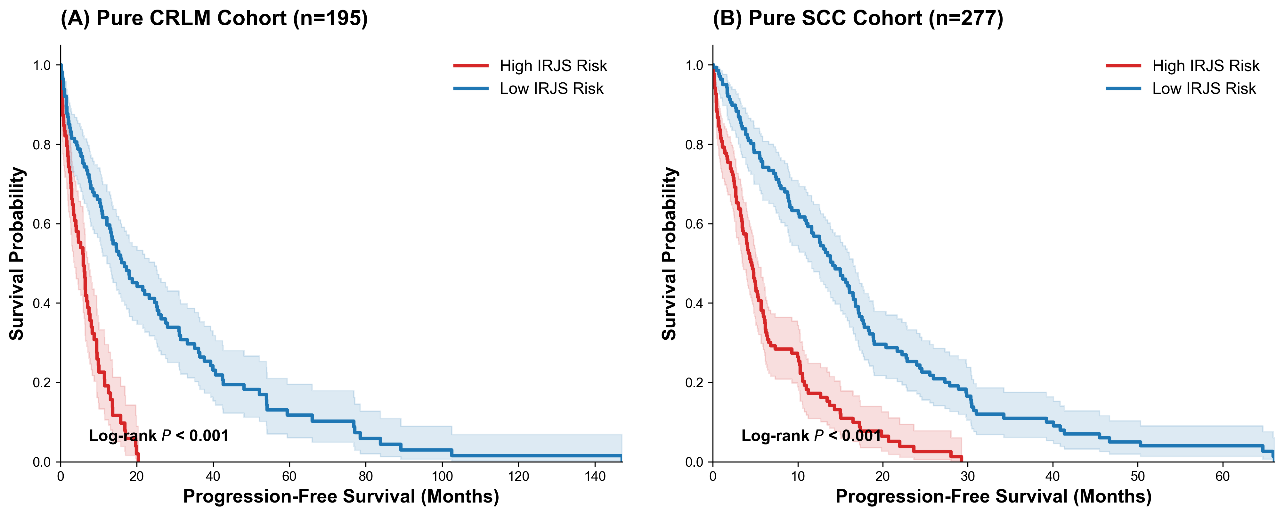


**Supplementary Figure S1.** Subgroup survival analysis validating the pan-cancer applicability of the TIME spatial barrier. Kaplan-Meier survival curves for progression-free survival (PFS) in the pure CRLM cohort (A) and the pure SCC cohort (B). High Immuno-Radiomics Joint Score (IRJS) consistently predicts inferior survival outcomes across distinct histological origins, confirming that the identified physical-metabolic spatial barriers act as a pan-cancer rule independent of primary histology. *P*-values were determined by the Log-rank test.


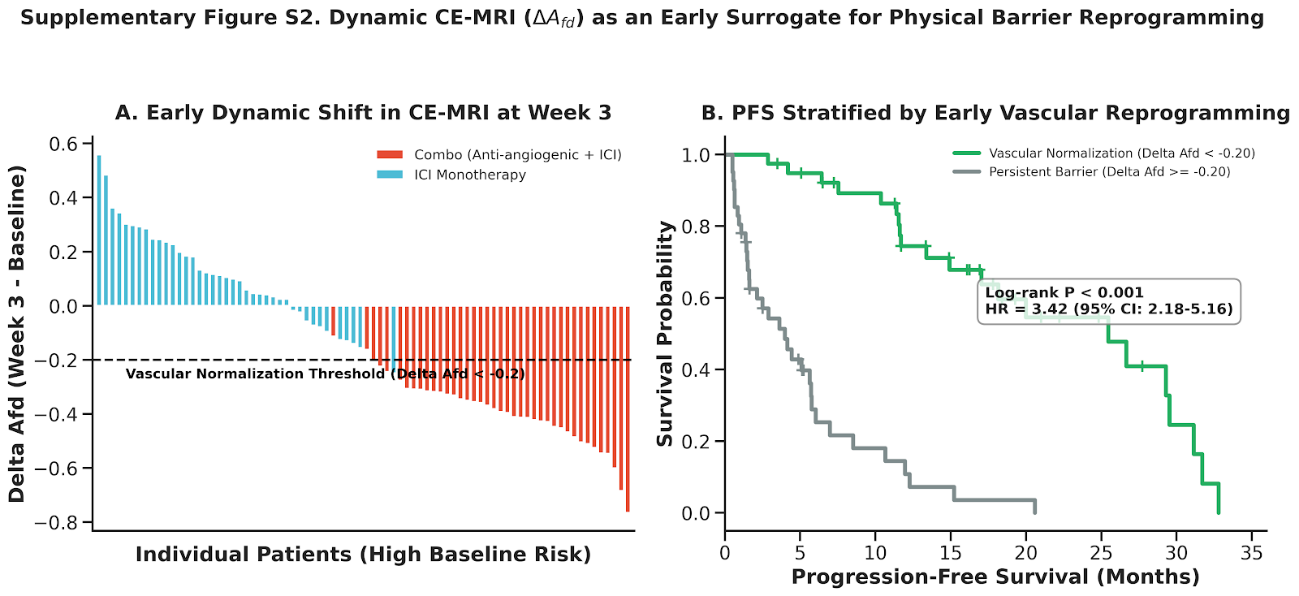


**Supplementary Figure S2.** Dynamic CE-MRI shift (∆A_fd) as an early surrogate for physical barrier reprogramming. (A) Waterfall plot illustrating the early dynamic shift in vascular fractal acceleration (∆A_fd) at week 3 in a refractory subset with high baseline IRJS risk (*n*=80). Responses are color-coded by treatment regimen (Combo vs. ICI Monotherapy). The horizontal dashed line represents the vascular normalization threshold ∆A_fd < -0.20), marking successful disruption of the physical barrier. (B) Landmark analysis (landmark set at week 3) of PFS stratified by early vascular reprogramming status. Patients achieving successful normalization exhibited significantly prolonged PFS compared to those with persistent barriers (∆A_fd ≥ -0.20). *P*-value was calculated by the Log-rank test.


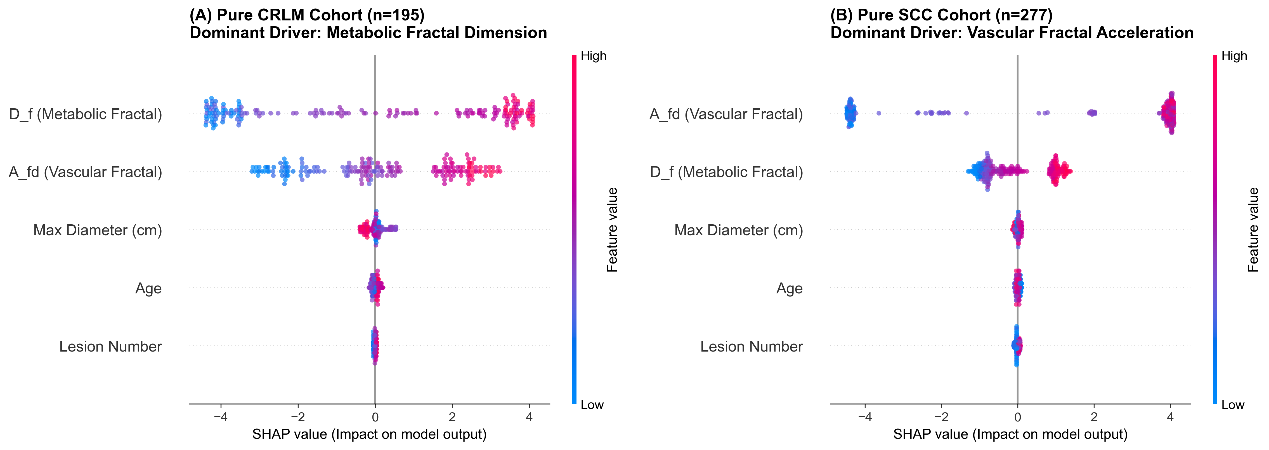


**Supplementary Figure S3.** SHAP summary plots demonstrating the robustness of fractal features in histology-specific sub-cohorts. (A) SHAP value distribution in the XGBoost model for the CRLM cohort (*n*=195), confirming metabolic fractal dimension (D_f) as the dominant driver. (B) SHAP value distribution in the SCC cohort (*n*=277), illustrating that vascular fractal acceleration (A_fd) maintains its top-ranking contribution. This sensitivity analysis effectively rules out shortcut learning, establishing A_fd and D_f as pan-cancer spatial immune parameters.


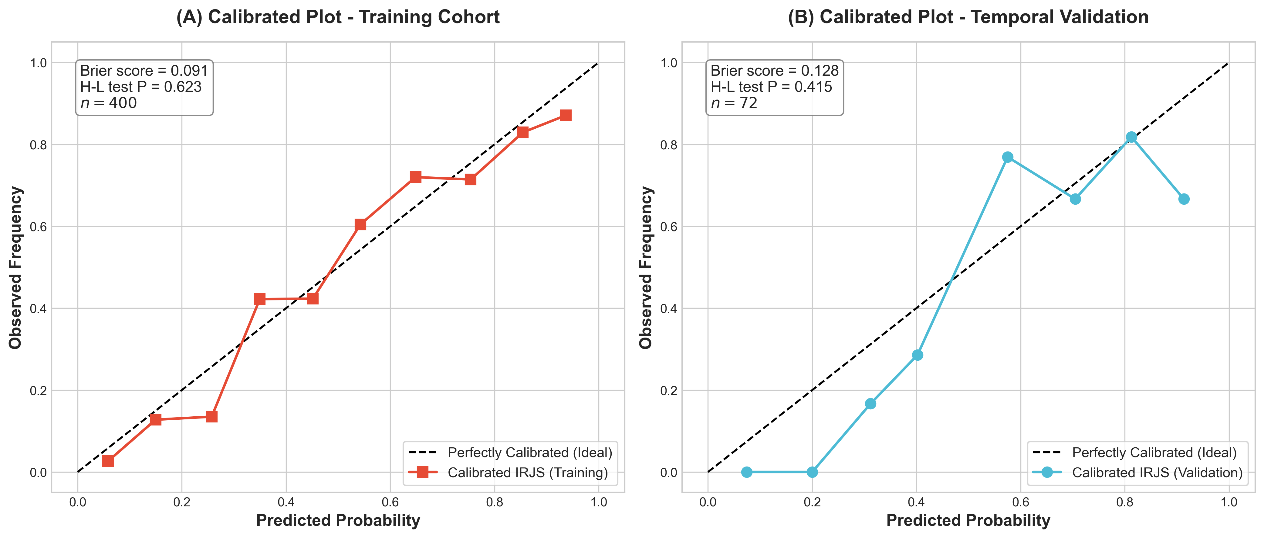


**Supplementary Figure S4. Calibration plots of the IRJS model after post-hoc Platt scaling.** (A) The training cohort (n=400) demonstrates excellent goodness-of-fit, closely tracking the 45-degree ideal line (Brier score = 0.091; Hosmer-Lemeshow test *P* = 0.623). (B) This robust calibration is successfully maintained in the independent temporal validation cohort (n=72, Brier score = 0.128; Hosmer-Lemeshow test *P* = 0.415).
