# Supplementary material for: Macroscopic fractal dynamics characterize the “physical-metabolic” dual barriers and systemic immune exhaustion associated with primary resistance to immunotherapy in liver metastases
Source: Front Immunol. 2026 Jul 9;17:1878195. doi: 10.3389/fimmu.2026.1878195 (PMC13391586; doi:10.3389/fimmu.2026.1878195)
Supplement: Supplementary file 5 [file DataSheet1.pdf]

# 锦州医科大学附属第一医院

# 生物样本库标本采集与使用知情同意书

**尊敬的患者/家属：** 您好！ 为了进一步推动医学科学的发展，探索疾病（特别是恶性肿瘤及其转移）的发生机制、早期诊断方法及精准治疗策略，锦州医科大学附属第一医院设立了标准化的“临床生物样本库”。我们诚挚地邀请您参与我们的生物样本捐赠计划。

在您决定是否同意捐赠之前，请仔细阅读以下内容。如果您有任何疑问，请随时向您的主管医生或研究人员提出。

**一、 标本采集的目的**

本生物样本库旨在收集、储存和管理各类临床生物样本，并将其用于经过本院独立临床研究伦理委员会严格审批的医学科学研究（如：探索肿瘤免疫微环境、肿瘤代谢特征、新型影像组学标志物及免疫治疗耐药机制等）。您的捐赠将为未来医学的发展和更多患者的治愈提供极其宝贵的资源。

**二、 标本采集的内容与方式**

如果您同意参与，我们将在不影响您正常临床诊疗的前提下，采集或保留以下样本：

1. **组织样本：** 在您进行常规穿刺活检或外科手术治疗时，在完全满足病理诊断需求且不影响您自身医疗利益的前提下，收集剩余的废弃病理组织。
2. **血液样本：** 在您进行常规临床抽血化验时，额外多抽取约 5-10 ml 的静脉血（用于分离血清、血浆或提取单个核细胞等）。
3. **临床及影像数据：** 允许研究人员在严格保密的前提下，从医院信息系统（HIS/PACS）中提取与您疾病相关的临床病历、病理报告及医学影像资料（如 CE-MRI、PET/CT 等）。

**三、 潜在风险与不适**

本标本采集计划带来的风险极小。组织样本的收集仅利用您常规诊疗后的剩余废弃物，不会增加任何额外的手术创伤或风险。血液样本的采集通常与您的常规抽血同步进行，极少数情况下可能会在抽血部位出现轻微疼痛、淤青或局部感染，医护人员将采取标准规范操作以将风险降至最低。

**四、 参与的获益与权益**

1. **直接获益：** 捐赠样本用于未来的医学研究，您个人可能不会从中获得直接的医疗或经济获益。但您的无私奉献将对医学科学的进步和未来其他患者的诊疗产生深远影响。
2. **知识产权与商业利益：** 基于您的样本所产生的科研成果（包括但不限于学术论文、专利、诊断试剂或新药研发等）、知识产权及潜在的商业利益，均归锦州医科大学附属第一医院或相关研究团队所有，不向捐赠者个人进行分配。

**五、 隐私保护与保密原则**

我们郑重承诺严格保护您的个人隐私。您的所有样本及临床数据在入库前均会进行**去标识化（匿名化）处理**，并分配唯一的专属条形码。在未来的研究和任何公开发表的学术成果中，均不会出现您的姓名、身份证号、联系方式等任何可识别您个人身份的信息。您的数据仅限于授权的研究人员在符合伦理规范的前提下使用。

**六、 自愿参与与随时退出权**

您的参与完全是**自愿的**。您有权拒绝参与本次标本捐赠，这绝对不会影响您在锦州医科大学附属第一医院获得的任何标准医疗服务、医患关系或任何既定权益。 即使您现在同意，您也有权在未来的任何时间改变主意并无条件退出。如果您决定退出，您可以联系您的主

管医生或生物样本库，我们将按照您的要求销毁您的剩余样本，并删除相关的临床研究数据（已经匿名化进入实质性研究阶段或已发表的数据除外）。

**患者/家属知情同意声明**

我已经阅读（或由他人向我宣读）了本《知情同意书》的全部内容，我的医生/研究人员已经向我详细解释了标本采集的目的、过程、风险及权益，并解答了我提出的所有问题。我完全理解本文件内容，并自愿同意将我的生物样本及相关临床资料捐赠给锦州医科大学附属第一医院生物样本库，用于经过伦理审批的医学科学研究。

**患者签名：** ________________ **日 期：** ______年___月___日

*（如患者因故无法亲自签名，由法定代理人或直系亲属代签）* **代签人签名：** ________________ **与患者关系：** ________________ **日 期：** ______年___月___日

---

**医生/研究人员声明**

我已向上述患者/家属充分解释了本次生物样本采集的目的、过程、风险、保密措施及患者享有的合法权益，并确认患者/家属在完全知情且自愿的情况下签署了本同意书。

**医生/研究人员签名：** ________________ **所在科室：** ________________ (如：核医学科)

**日 期：** ______年___月___日

锦州医科大学附属第一医院
医学研究伦理委员会
